# Deep generative modeling for volume reconstruction in cryo-electron microscopy

**DOI:** 10.1016/j.jsb.2022.107920

**Published:** 2022-11-08

**Authors:** Claire Donnat, Axel Levy, Frédéric Poitevin, Ellen D. Zhong, Nina Miolane

**Affiliations:** a University of Chicago, Department of Statistics, Chicago, IL, USA; b Stanford University, Department of Electrical Engineering, Stanford, CA, USA; c LCLS, SLAC National Accelerator Laboratory, Menlo Park, CA, USA; d Princeton University, Department of Computer Science, Princeton, NJ, USA; e University of California Santa Barbara, Department of Electrical & Computer Engineering, Santa Barbara, CA, USA

**Keywords:** cryoEM, Deep neural networks, Generative models, High-resolution volume reconstruction

## Abstract

Advances in cryo-electron microscopy (cryo-EM) for high-resolution imaging of biomolecules in solution have provided new challenges and opportunities for algorithm development for 3D reconstruction. Next-generation volume reconstruction algorithms that combine generative modelling with end-to-end unsupervised deep learning techniques have shown promise, but many technical and theoretical hurdles remain, especially when applied to experimental cryo-EM images. In light of the proliferation of such methods, we propose here a critical review of recent advances in the field of *deep generative modelling for cryo-EM reconstruction*. The present review aims to (i) provide a unified statistical framework using terminology familiar to machine learning researchers with no specific background in cryo-EM, (ii) review the current methods in this framework, and (iii) outline outstanding bottlenecks and avenues for improvements in the field.

## 1. Introduction

**Fig. 1.**
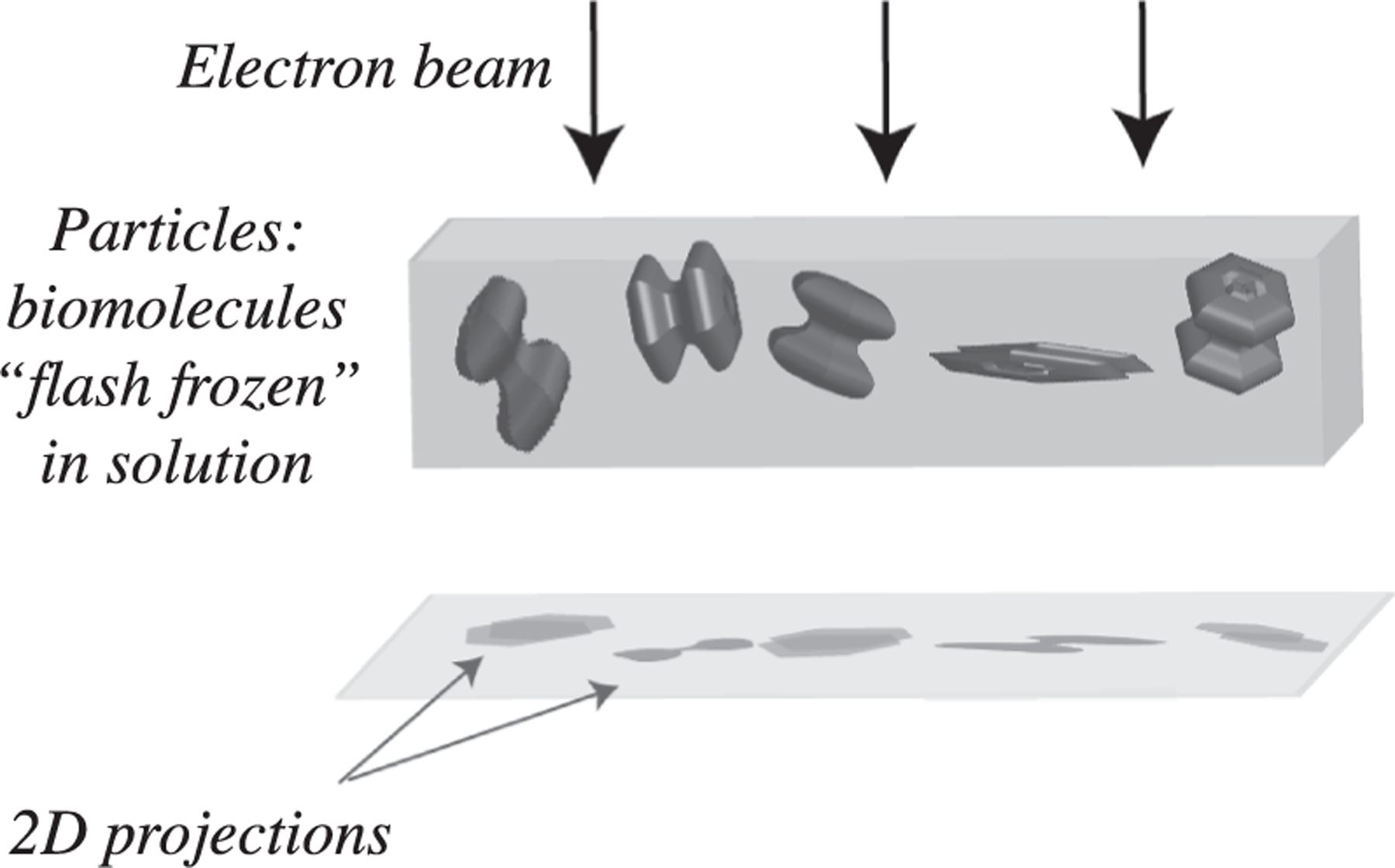
Schematic of single particle cryo-EM. Acquisition of 2D cryo-EM images (2D projections) from 3D biomolecular volumes, flash frozen in an unknown orientation.

Advances in cryo-electron microscopy (cryo-EM) for high-resolution imaging of biomolecules in solution have driven a revolution in structural biology (Nakane et al., 2020; EMDB, 2022), facilitating breakthroughs in our ability to understand fundamental biological mechanisms and engineer macromolecular function (Ourmazd, 2019; Renaud et al., 2018). However, the estimation of the imaged molecules’ 3-dimensional (3D) structure from cryo-EM imaging data poses a major computational challenge. In single particle cryo-EM, molecules have been flash-frozen in a thin layer of vitreous ice; raw observations from the microscope are limited to the 2D projections of individual molecules (also called particles) relative to an incoming electron beam (Fig. 1). The resulting cryo-EM reconstruction task aims to recover the unknown 3D volumes of the imaged molecules from their 2D projections, a challenging inverse problem complicated by the unknown pose of each particle relative to the electron beam, the variability in the shape of any given molecule (also referred to as structural heterogeneity), the non-linear physics of the data acquisition process, as well as extremely low signal-to-noise ratios.

Reconstruction algorithms are typically formulated as the statistical estimation of an underlying 3D density volume along with additional unknown latent variables, such as the image pose. Reconstructing molecular volumes thus becomes a highly non-convex optimization problem, putting algorithms at risk of being overly sensitive to initialization and converging to local minima (Boyd et al., 2004). The difficulty of this task is further compounded by the fact that each molecule has its own unknown conformation (or shape). Methods that account for this heterogeneity are called **heterogeneous reconstruction methods**. While a challenge for 3D reconstruction, this heterogeneity presents a major advantage of cryo-EM relative to other approaches in structural biology, such as X-ray crystallography, that produce ensemble averages. Furthermore, advances in the microscope automation are resulting in much larger datasets; modern algorithms can take advantage of the increased data to improve resolution and resolvability of structures, yet they must overcome the associated computational challenges in dealing with large amounts of imaging data. In this context, recent efforts have turned to unsupervised deep learning for cryo-EM reconstruction. These approaches present new opportunities to model heterogeneity through expressive deep neural network architectures, enabled through the use of gradient-based optimization and GPU compute. Given their potential to advance the field by addressing the challenges mentioned here, we propose here a critical review of deep generative modeling for cryo-EM volume reconstruction.

Our objective is to overview the similarities and differences among recent state-of-the-art, deep-generative reconstruction methods, which we classify according to *(i) their parametrization of the generative model* (Section 2) and *(ii) the inference tools* deployed to fit this generative model (Section 3). This unification of recent works along a consistent statistical framework allows us to highlight trends, outstanding challenges, and avenues for improvements in the field (Section 4). The reviews by Singer et al. (2020) and Bendory et al. (2020) provide a complete description of cryo-EM reconstruction, but focus on mathematical foundations of general computational methods, rather than specifically on deep learning approaches. Reviews by Si et al. (2021), Ede (2021) and Wu et al. (2021) describe applications of deep learning methods along all steps of the cryo-EM pipeline, without specialising to 3D volume reconstruction. By contrast, our review is a deep dive into the theory and methods of recent deep generative models for cryo-EM reconstruction. Through this review, we hope to catalyze deep learning advances by providing machine learning practitioners and computer vision experts a thorough overview of the challenges that are unique to cryo-EM.

## 2. Generative Modeling for Cryo-EM

The objective of cryo-EM imaging algorithms is to produce a 3D reconstruction of a given molecule from a dataset of images ${\left\{X_{i}\right\}}_{i=1\cdots n}$, where each image corresponds to a “2D projection” of a unique instance of the molecule at a different (unknown) pose (Fig. 1). A major design choice in reconstruction algorithms involves the parameterization of the volume and the conformational space for modeling heterogeneity. This section reviews the trade-offs in these choices, their inductive biases, and how they yield different formulations of the cryo-EM image formation model.

### 2.1. Image formation model

The process of image formation in cryo-EM involves several physical phenomena, including pairwise interactions between atoms, interactions between the electron beam and the molecule’s electrostatic potential, and microscope effects. We refer the reader to Dill et al. (2010), Kohl and Reimer (2008), and Vulović et al. (2013) for in-depth descriptions of these phenomena. Nonetheless, in most cases (Scheres, 2012; Vulović et al., 2013), each image $X_{i}$ in a dataset of n images of single particles can be modeled as a random sample from the following generative model:

(1) $$X_{i}={\text{PSF}}_{i}*\left(t_{i}\circ {\text{Π}}_{2D}\circ R_{i}\right)\left(V^{\left(i\right)}\right)+\in_{i}\;,\;\text{for }i=1\cdots n.$$

Here, $R_{i}$ is a 3D rotation representing the 3D orientation of the volume $V^{\left(i\right)}$ with respect to the direction of the electron beam. The oriented volume is subsequently “pierced through” by the electron beam and projected onto the detector — an operation represented in Eq. (1) by the 2D-projection operator $\Pi_{2}D$. The variable $t_{i}$ represents the 2D translation of the projected volume with respect to the center of the image. The effect of the microscope’s lens is modeled through the convolution ∗ of the 2D projection by an image-dependent operator ${\text{PSF}}_{i}$ called the Point Spread Function (PSF) of the microscope whose parameters can depend on the image. We note here that an initial estimate PSF, shared across images i from a given acquisition (called “micrograph”), is usually computed before reconstruction, and then refined as an estimate of ${\text{PSF}}_{i}$ per image. Finally, additional noise $\in_{i}$ is introduced in the observed image, and typically assumed to be Gaussian with zero mean and variance $\sigma_{i}^{2}$. Note that the underlying volume $V^{\left(i\right)}$ is allowed to depend on i. This allows us to account for “conformational heterogeneity”, a concept whereby a molecule does not necessarily exist in a single state, but rather, that its volume corresponds in fact to one of several stable geometries that can be achieved by this molecule.

An equivalent generative model can be formulated via the Fourier transform of Eq. (1) which we present in the supplementary material. In Fourier Space, the convolution ^∗^ of the projected volume by the PSF becomes a computationally lightweight element-wise matrix multiplication ⊙ between the 2D Fourier transform of the projected image and that of the PSF (known as the *Contrast Transfer Function*, or *CTF*). Operating in Fourier space is thus common in many cryo-EM volume reconstruction algorithms.

### 2.2. Conformation variable z.

Heterogeneous reconstruction methods introduce an additional variable $z_{i}$ for each image i within the formation model of Eqs. (1), which we call the conformation variable. Depending on whether conformation heterogeneity is modeled through a discrete number of states or as a continuous variable, the conformational landscape can be encoded as a discrete family of volumes $V=\left\{V_{z},\;z\in \left\{1,\ldots ,\;K\right\}\right\}$ **(discrete heterogeneity)**, or as a continuous family $V=\left\{V\left(z\right),\;z\in {\mathbb{R}}^{L}\right\}$ for some integer L **(continuous heterogeneity)** (Jonić, 2017). In both cases, the family is indexed by the variable z. We use the notations $V_{z}$ and $V\left(z\right)$ interchangeably. Homogeneous reconstruction can in fact be taken as the special case where V only comprises a single volume, so that K=1 or L=0 (i.e. forcing z=0). We now write $V^{\left(i\right)}=V\left(z_{i}\right)$ in Eq. (1).

#### Interpretation of the Conformation Variable

From a statistical mechanics perspective, the conformation variable $z_{i}$ encodes the location of any given single particle along the conformational landscape (Dill et al., 2010). For example, if $z_{i}\in \mathbb{R}$, $z_{i}$ can be used to sort conformations along a “reaction coordinate”, that is, a sequence of small transformations that would interpolate two main preferred, dynamically stable states. When continuous, the dimension L of this cursor variable z could in principle take any value between 0 (no heterogeneity) and $O\left(N\right)$, with N the number of atoms in the molecule. However, two factors tend to drastically limit the number of dimensions of z. First, most of the main global dynamics of a molecule are captured by a few collective variables associated with its low-frequency motion, effectively averaging out a lot of the effects of the high number of degrees of freedom associated with faster motions (Noe and Clementi, 2017). Second, limitations of imaging apparatus, as well as imprecision in the determination of the point spread function PSF often reduce the ability to resolve the remaining motions, thus reducing the effective dimensionality of z (Katsevich et al., 2015). In other words, limits in the imaging technology itself restrict the dimension of the variable z. In the case of discrete heterogeneity, $z\in \left\{1,\ldots ,\;K\right\}$ is an index of minimum energy wells (conformations) in the conformational landscape. Imaging conditions also reduce the ability to resolve too many metastable states, thereby restricting practitioners to choose a low value for K. Finally, we note that the interpretation of z should be performed in the context of the parameterization of the molecular volume (Section 2.3).

#### Discrete vs Continuous Conformational Heterogeneity: Pros, Cons and Discussion.

Discrete heterogeneity has a rich history in cryo-EM. Popularized by the ”3D classification” (Scheres, 2012; Scheres, 2010) extension of RELION (Scheres, 2012), it offers the advantage of delivering readily interpretable results: a set of K volumes, representing K main stable states of the molecules. Discrete heterogeneity is thus particularly adequate in certain (common) scenarios where the conformation landscape has local energy minima that produce distinct states. However, one of the main drawbacks of this method consists in the necessary selection of the number K of appropriate conformations. Theoretically, this could be done by cross-validation. In practice, due to the significant computing costs that cross-validation implies, K is chosen in an ad hoc fashion by the experimenter and rarely motivated by strong quantitative arguments (see Haselbach et al. (2018) for a rare example). Furthermore, the final reconstructed volumes are severely biased by the initialization of the K volumes, leading to very ad-hoc tuning procedures.

Consequently, many recent methods have turned to a continuous representation of heterogeneity which does not require specifying a number K of conformations. This representation is also often deemed to be closer to the underlying biology, as molecules do not exist as finite/discrete sets of shapes. Rather, a more realistic analogy is to think of molecules as random samples from the equilibrium distribution over their conformational space (Dill et al., 2010). However, while a continuous representation could be more scientifically relevant, it remains to be determined how accurate the reconstruction of the conformational space by the space indexed by z truly is. This latter point will be critical to address for heterogeneous reconstruction methods to become more quantitative and directly comparable to other measures from biophysicists and biochemists. We discuss in Section 4 the challenges of assessing the precision of such approaches, which probably constitutes one of the main open questions in the field. Additionally, despite its initial appeal, continuous conformation heterogeneity comes with significant theoretical and practical caveats. From a physics perspective, it is still unclear whether the full landscape (at room temperature) is sufficiently well sampled by cryo-EM to justify modeling conformations with a continuous rather than discrete distribution: the sample preparation process in cryo-EM — and most specifically the grid-freezing step—affects the distribution of conformations which might not reflect the heterogeneity of conformations at room temperature (Bock and Grubmüller, 2021). From a statistical perspective, using a continuous distribution necessitates the generative model to be able to sample from the full conformation landscape, a requirement that is itself a considerable challenge for large molecules: the strong constraints, e.g. on bond lengths and torsion angles, make up for a complex, non-convex landscape that is difficult to sample from. Despite these caveats, Fig. 3 shows that continuous heterogeneity is gaining traction amongst the most recent reconstruction advances.

### 2.3. Molecular volume $V\left(z\right)$

The heterogeneous cryo-EM reconstruction problem can thus be understood as recovery of the underlying conformational landscape V and the corresponding probability distribution. The next critical step thus consists of choosing a parametrization for each volume $V\left(z\right)\in V$. This requires choosing first an “input domain” (continuous vs. discrete), second, an “output space” (image space vs. Fourier space, inducing real vs complex values), and third, an “encoding style” (reference-free vs. reference-based).

#### 2.3.1. Defining the input domain: discrete or continuous

The volume $V\left(z\right)$ represents a scalar 3D field (i.e. an electrostatic potential, or its Fourier transform) and is defined as a function from the input domain $\text{Ω}\subset {\mathbb{R}}^{3}$ to an output space ℝ (or ℂ). We now describe the parametrization of Ω and distinguish two cases, depending on whether the volume is defined as a discretized or as a continuous scalar field.

##### Discretized Domain and Explicit Parametrization.

The first class of approaches models the electrostatic potential as a discrete 3D map. In this case, the function $V\left(z\right)$ is defined on a discretized subspace (a grid) of ${\mathbb{R}}^{3}$, namely $\text{Ω}={\left\{1,\ldots ,\;D\right\}}^{3}$, where D represents the length of the 3D voxel grid or frequency grid. $V\left(z\right)$ is explicitly parametrized by the values it takes at each location (or voxel) of Ω. This choice is also called an *explicit* parametrization, a term that will become clear in the next paragraph. Using a vectorial formalism, the vector $V\left(z\right)$ corresponds to voxels’ intensity values, with $V\left(z\right)\in {\mathbb{R}}^{D^{3}}$ or $\in {\mathbb{C}}^{D^{3}}$. In this case, the resolution of the reconstructed volume is fixed by the choice of the granularity of the grid. However, the vectorial formalism would imply that $V\left(z\right)$ becomes an infinite-dimensional vector when it is represented continuously (see next paragraph). For this reason, we prefer to use a functional formalism and define the volume $V\left(z\right)$ as a function (not as a vector), whether it is modeled as a discrete or continuous field. Discrete domains are adopted by methods like RELION-Refine3D (Scheres, 2012) and RELION-Class3D (Scheres, 2012) — which associate voxels with corresponding intensities in Fourier space—, and like CryoPoseNet (Nashed et al., 2021) or 3DFlex (Punjani and Fleet, 2021) in image space.

##### Continuous Domain and Implicit Parametrization.

The second class of methods model the volume $V\left(z\right)$ as a continuous field, *i.e.* as a function on a continuous domain ($\text{Ω}={\mathbb{R}}^{3}$ or $\text{Ω}={\left[-0.5,\;0.5\right]}^{3}$). The domain Ω is infinite, and one cannot explicitly maintain in memory the values that $V\left(z\right)$ takes on Ω. The solution is then to adopt an explicit parametrization for $V\left(z\right)$ using parameters $\theta \in \text{Θ}\in {\mathbb{R}}^{p}$. Depending on whether or not these parameters have a physical meaning (*e.g.* centroids of pseudo-atoms), the function $V\left(z\right)$ can be encoded:

- **Using Neural Networks.** Some methods use neural networks to represent $V\left(z\right)$ as a (real or complex) function of a 3D position vector. The parametrization is called “implicit” because the values of $V\left(z\right)$ are not stored in memory; instead, the practitioner can “query” the neural network by inputing any location $x\in {\mathbb{R}}^{3}$ and receiving a value for $V\left(z\right)$ at x. In this case, the parameters θ — *i.e.* the weights of the neural network — do not have a physical meaning. Examples of this approach include CryoDRGN (Zhong et al., 2019) and CryoAI (Levy et al., 2022), both operating in Fourier space using a reference-free volume encoding.
- **Using Gaussian Mixtures.** Other approaches constrains the volume $V\left(z\right)$ by modeling the source of the electrostatic potential: its individual atoms or pseudo-atoms. Indeed, at a granular level, the molecular volume can be approximated by a mixture of N Gaussian functions (called scattering form factors (Kohl and Reimer, 2008)) of the form:
(2) $$V_{z}\left(x\right)=\sum_{j=1}^{N}A_{j}\;\text{exp}\left(-\frac{{\left\|c_{j}-x\right\|}^{2}}{2\sigma_{j}^{2}}\right),$$
  where $x\in {\mathbb{R}}^{3}$ represents a 3D position, and $c_{j}\in {\mathbb{R}}^{3}$ are the 3D coordinates of the N individual atoms or pseudo-atoms. The parameters $A_{j}\in \mathbb{R}$ and $\sigma_{j}^{2}\in \mathbb{R}$ describe how each (pseudo-) atom contributes to the electrostatic potential. In practice, these approaches always implement conformational heterogeneity, and do so through a continuous conformation variable $z\in {\mathbb{R}}^{L}$ that passes through a neural network to output $c_{j}$, and possibly $A_{j}$ and $\sigma_{j}^{2}$. This approach also models $V_{z}$ as a continuous field, as defined by Eq. (2), but the parameters defining each volume $\left(\theta ={\left\{c_{j},\;A_{j},\;\sigma_{j}^{2}\text{)}\right\}}_{j=1,\ldots ,\;J}\right)$ now have a physical meaning. Among this general class of methods, works differ in whether $A_{j}$, $\sigma_{j}^{2}$ are assumed to be known, and in the interpretation given to the variable $c_{j}$. E2GMM (Chen and Ludtke, 2021) uses a conformation variable z that encodes the coefficients $c_{j}$, $A_{j}$, $\sigma_{j}^{2}$ and defines the $c_{j}$ as coordinates of “coarse grained atoms” (reference-free). CryoFold (Zhong et al., 2021) assumes $A_{j}=A$ and $\sigma_{j}=\sigma$ known and fixed while using $c_{j}$ to represent “groups of atoms”. AtomVAE (Rosenbaum et al., 2021) also assumes and $\sigma_{j}=\sigma$ known and fixed, models the $c_{j}$ as the coordinates of the atoms, and uses the conformation variable z to encode heterogeneous deviations $\text{Δ}c_{j}$.

##### Discretized and Continuous Domains: Pros, Cons and Discussion.

Contrary to the discretized domains, approaches using continuous domains potentially allow to achieve sharper, enhanced resolutions (within the Nyquist limit), as any coordinate of ${\mathbb{R}}^{3}$ can be fed to $V\left(z\right)$. Moreover, within continuous approaches, pseudo-atomic methods effectively add constraints to $V\left(z\right)$ by modeling it as a mixture of Gaussians, and even more so when assuming a reference conformation $V_{0}$. The increasing availability of folded protein shapes — traditionally from the Protein Data Bank (Rose et al., 2021) and more recently through the advent of AlphaFold (AlQuraishi, 2019) — have indeed enabled access to relatively reliable atom coordinates of reference conformations $V_{0}$, that can enrich the recovery of the molecular volume. We also note that reference-based representation such as that proposed in AtomVAE (Rosenbaum et al., 2021) and CryoFold (Zhong et al., 2021) are more amenable to the inclusion of molecular dynamics information to the volume reconstruction process.

#### 2.3.2. Defining the output space: image space or Fourier space

The image formation model can be described equivalently in image space or Fourier space, as shown by Eq. (1) and its corresponding Fourier formulation provided in the supplementary material. Thus, each volume within the family of conformations V can be described either in terms of its pixel intensities or its Fourier coefficients. In either case, the volume $V\left(z\right)$ associated to the conformation variable z is defined on an input domain $\text{Ω}\subset {\mathbb{R}}^{3}$ (the space of coordinates) and outputs values in an output space that is either ℝ for pixel intensities representing the electron scattering potential of the molecule, or ℂ to encode the amplitude and phase of the Fourier coefficients.

##### Image versus Fourier space: Pros, Cons and Discussion.

From a practical standpoint, the choice of the output space is guided by the set of properties and constraints that the analyst wishes to use to guide volume reconstruction. Historically, the Fourier approach has been preferred. As summarized by Punjani and Fleet (2021), working in Fourier space has the benefits of (a) reducing the computational cost of the image formation model (see discussion of the generative model in Fourier space), and (b) allowing closed-form maximum likelihood reconstructions when molecules’ orientations and positions are known. However, recent methods such as 3DFlex (Punjani and Fleet, 2021) have favored image space, where constraints (e.g. smoothness of the deformation, conservation of energy, etc.) are more interpretable and where operations such as interpolation and deformation of the molecule’s density map are more naturally parametrized — whereas the same operations require a careful treatment in Fourier space. For example, interpolation in Fourier space can introduce unwanted artifacts. As highlighted in Fig. 3, image space computations constitute a promising and increasingly popular avenue for future developments in cryo-EM reconstruction.

#### 2.3.3. Defining an encoding: reference-free or reference-based volume

Finally, different algorithms typically make a choice of an “encoding” for the volume $V\left(z\right)$ either *(i) using a reference-based parametrization*, which encodes the conformation landscape through its deviation $\Delta V\left(z\right)$ from a reference conformation $V_{0}$, such that $V\left(z\right)=V_{0}+\text{Δ}V\left(z\right)$; or *(ii) using a reference-free parametrization* which directly describes each $V\left(z\right)$, for instance as a set of atomic coordinates or a low-dimensional embedding, but with no notion of “reference” conformation.

##### Reference-based versus reference-free: Pros, Cons and Discussion

If the Fig. 3 reflects the historical popularity of reference-free encodings, the most recent methods relying on deep-learning seem to have favored a reference-based approach. For instance, E2GMM (Chen and Ludtke, 2021) first learns a reference $V_{0}$ called the “neutral representation” which then serves in a reference-based encoding of $V\left(z\right)$ to further refine the reconstruction by accounting for conformational variability. In AtomVAE (Rosenbaum et al., 2021), Rosenbaum et al. uses a $V_{0}$ called a “base conformation” described as a set of atom coordinates obtained from an auxiliary method, such as an homogeneous reconstruction or a set of atom coordinates predicted by AlphaFold (AlQuraishi, 2019). The existing reference acts as a statistical prior on the molecular volume, thereby further constraining and guiding the recovery of the conformation landscape. By contrast, 3DFlex (Punjani and Fleet, 2021) uses a reference volume $V_{0}$, called a “canonical density”, which is learned jointly with the conformational heterogeneity. This has the advantage of foregoing the need to split the pipeline in sequential steps, while allowing to borrow strength from the joint estimation of all parameters.

Constraining the conformation recovery using a reference offers significant advantages for ensuring the success (and convergence) of these methods given the non-convexity of the problem. This template can be either learned (ab initio methods), or chosen from existing data (refinement methods — more on this in the supplementary material). The general agreement across all methods consists in tackling this hierarchically, starting with parameters which have the strongest impact on the signal, such as defocus or pose, and gradually focusing on those whose effect is more subtle, such as local deformations. As such, biasing the solution V towards a reference $V_{0}$, such that $\text{Δ}V\left(z\right)=0$ implies $V\left(z\right)=V_{0}$, can provide an interesting way of ensuring a more reliable and consistent — but potentially biased — solution. Depending on the optimization method used, this can in fact be critical to the success of the pipeline: Rosenbaum et al. (2021) report that adopting a reference template and warm-starting their algorithm is indispensable to ensure the recovery of good conformations. However, because they fundamentally bias conformations $V\left(z\right)$ to “hover” around $V_{0}$, the success of such methods necessitates a reliable $V_{0}$. This can also incur higher computational costs, since such methods typically require running a first reconstruction method. This explains the interest for alternative, reference-free methods: three out of the six heterogeneous methods in Fig. 3 allow to recover molecular volumes without any prior template. The extent to which these reference-free methods are likely to succeed on real-images still remains to be characterized.

## 3. Inference

We now turn to the description of the inference methods used in deep generative modeling for cryo-EM reconstruction. These methods recover the volume V by finding optimal parameters θ, conformation variables $z_{i}$ and nuisance variables (${\text{PSF}}_{i}$, $R_{i}$, $t_{i}$) of the generative model in Eq. (1). In this section, θ collectively denotes the parameters that describe the conformational landscape as a function of z, and the parameters of the function $V_{z}\;\text{:}\;x\to \text{Ω}$ that associates a position x to an output intensity. We refer to the conformation variable and poses jointly as the “hidden variables” and denote them as $H_{i}=\left(z_{i},\;{\text{PSF}}_{i},\;R_{i},\;t_{i}\right)$. This section overviews general inference methods with a description of their variations given in the supplementary material.

### Setting Up the Inference Problem: Observed Likelihood vs Full Likelihood

In the context of deep generative modelling for cryoEM, the cornerstone of inference is simply the observed likelihood $p_{\theta }\left(x\right)=p\left(x\text{|}\theta \right)$ associated with each image x. This likelihood is computed from the generative model in Eq. (1) (or its Fourier counterpart provided in the supplementary material), which we seek to maximize as a function of θ. However, the generative model depends on hidden variables $H_{i}=\left({\text{PSF}}_{i},\;t_{i},\;R_{i},\;z_{i}\right)$. In most cases, the optimization of the full likelihood of each observation $p\left(x_{i},\;h_{i},\;\theta \right)$ would be quite simple, if only the $H_{i}$ were observed. Thus, given n observed images $x_{1},\ldots ,\;x_{n}$, one solution could be to jointly recover the parameters θ and hidden variables H (considered here as fixed quantities, as opposed to random variables) that maximize the log-likelihood $\ell \left(X,\;\theta \right)=\sum_{i=1}^{n}\text{log}\left(p_{\theta }\left(x_{i},\;h_{i}\right)\right)$. Mathematically, this requires solving the following optimization problem:

(3) $$\theta^{*},\;H^{*}={\text{argmax}}_{\theta ,\;H}\sum_{i=1}^{n}\text{log}\left(p_{\theta }\left(x_{i},\;h_{i}\right)\right)$$

It is in fact a classical exercise in statistics to show that in this case, as the number of estimated variables grows with the number of data points, the estimate of θ is no longer guaranteed to converge to the true underlying value as n goes to infinity: ${\text{lim}}_{n\to \infty }E\left[\theta^{*}\right]\neq \theta^{\text{true}}$. We thus have to resort to strategies that treat hidden variables as random variables, and that fit the parameters θ based on the “observed likelihood” $L\left(X,\;\theta \right)$. In this case, the objective becomes:

(4) $$\theta^{*}={\text{argmax}}_{\theta }L\left(X,\;\theta \right)\;\text{where}\;L\left(X,\;\theta \right)=\sum_{i=1}^{n}\text{log}p\left(x_{i}\text{|}\theta \right)\;\;=\sum_{i=1}^{n}\text{log}\int_{h_{i}}p\left(x_{i},\;h_{i}\text{|}\theta \right)d\mu \left(h_{i}\right)$$

where $d\mu \left(h\right)=p\left(h\right)dh$ is the probability measure associated to the hidden variables H. However, this marginal likelihood requires an integral over all possible values of $H_{i}$. This quantity is difficult to compute directly, or in statistical terminology, “intractable”. Consequently, the crux of the optimization pipeline is to find a way to effectively approximate it.

### 3.1. Unifying inference methods

Since the observed log-likelihood $L\left(X,\;\theta \right)$ in the objectives of Eq. (4) (and its maximum a posteriori version provided in the supplementary material) is intractable, optimization is usually performed by targeting a proxy for $L\left(X,\;\theta \right)$, called the Evidence Lower Bound (ELBO). For the sake of clarity and concision, we highlight here the common statistical thread of cryo-EM reconstruction methods leveraging deep generative modeling, that all use an ELBO-based optimization and refer the reader to the supplementary material for further discussion on their variations.

#### Evidence Lower Bound (ELBO)

**Fig. 2.**
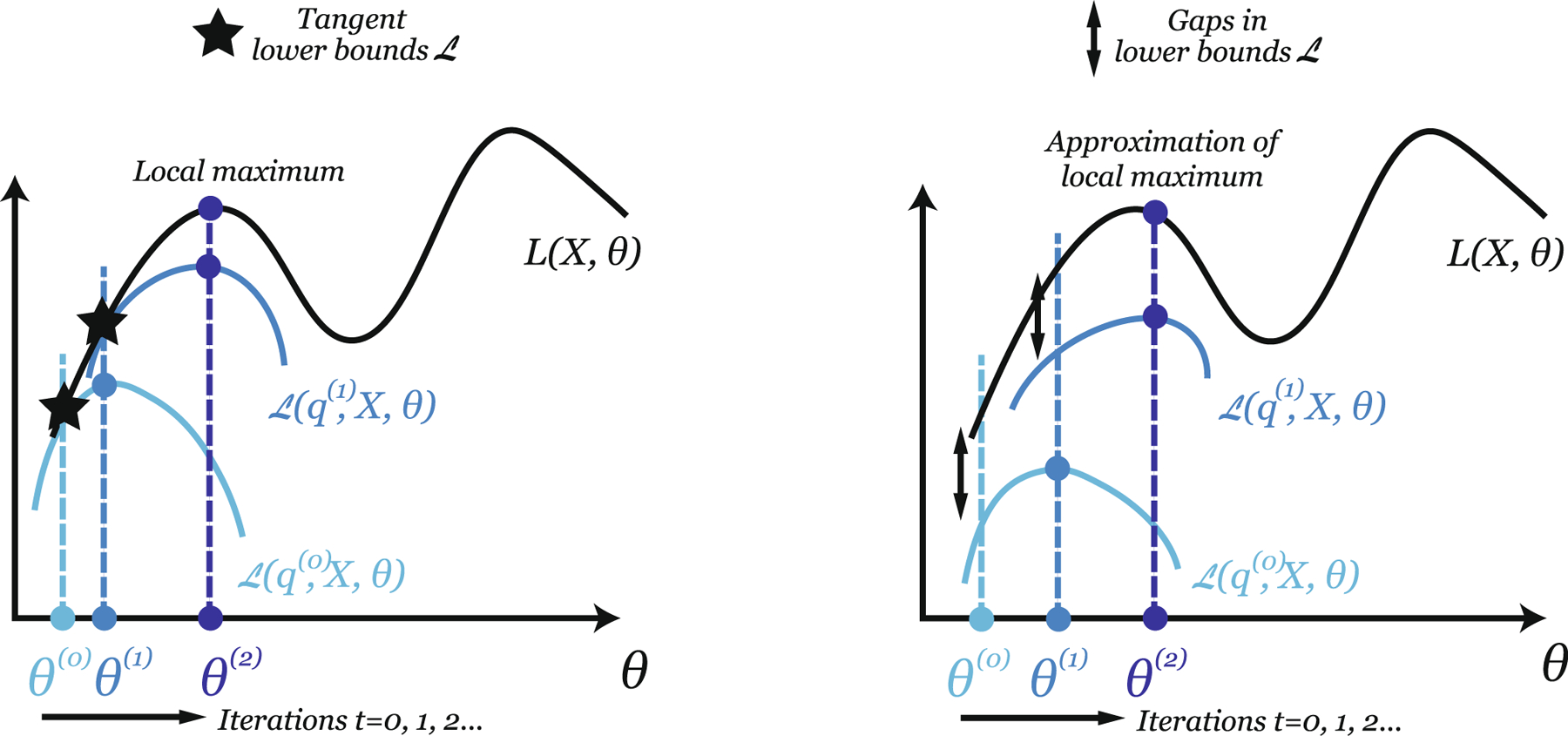
Maximization of the log-likelihood $\theta \to L\left(X,\;\theta \right)$ in θ by maximizations of a series of lower bounds: $L\left(q^{\left(0\right)},\;X,\theta \right)$, $L\left(q^{\left(1\right)},\;X,\theta \right)$, etc. The $\theta^{\left(t\right)}$ s across iterations t=0, 1, 2,…are represented by colored dots and correspond to successive maxima of the lower bounds. Left: The lower bounds are tangent to $\theta \to L\left(X,\;\theta \right)$, which is realized when q is the posterior of the hidden variables. Right: The lower bounds are not tangent to $\theta \to L\left(X,\;\theta \right)$, but show a “gap” that corresponds to the KL divergence between q and the posterior of the hidden variables, see Eq. (5).

The trick behind the Evidence Lower Bound (ELBO) consists in proposing a series of distributions $q^{\left(0\right)},\ldots ,\;q^{\left(t\right)}$ for the hidden variables H, and maximizing a series of “easily” computable lower-bounds $L\left(q^{\left(0\right)},\;X,\;\theta \right),\ldots ,\;L\left(q^{\left(t\right)},\;X,\;\theta \right)$ for $L\left(X,\;\theta \right)$ in an iterative fashion — see Fig. 2. By iteratively maximizing these lower bounds with respect to θ, the true likelihood $L\left(X,\;\theta \right)$ also increases. The hope is that the value of θ obtained through their maximization will be close to the value realizing the maximum of $L\left(X,\;\theta \right)$, if the lower bounds are tight enough — *i.e.* for small “gaps” in Fig. 2.

The lower bounds $L\left(q,\;X,\;\theta \right)$ are found by showing that, for any probability distribution $q_{i}$ on the variables $h_{i}$, the observed log-likelihood can be written as the sum of two terms (derivations provided in the supplementary material):

(5) $$L\left(X,\;\theta \right)=L\left(q,\;X,\;\theta \right)+\sum_{i=1}^{n}\text{KL}\left(q_{i}\left(h_{i}\right)∥p_{\theta }\left(h_{i}\text{|}x_{i}\right)\right)\;\;\;\;\;\;=\sum_{i=1}^{n}\left[L_{i}\left(q_{i},\;x_{i},\;\theta \right)+\text{KL}\left(q_{i}\left(h_{i}\right)∥p_{\theta }\left(h_{i}\text{|}x_{i}\right)\right)\right]$$

where KL is the Kullback–Leibler divergence $\left(\text{KL}\right)$ defined as $KL\left(q∥p\right)=\int q\left(x\right)\text{log}\frac{q\left(x\right)}{p\left(x\right)}dx$, and the terms $L_{i}$ write:

(6) $$L_{i}\left(q_{i},\;x_{i},\;\theta \right)=\int_{h_{i}}q_{i}\left(h_{i}\right)\text{log}p_{\theta }\left(x_{i}\text{|}h_{i}\right)dh_{i}-\text{KL}\left(q_{i}\left(h_{i}\right)∥p_{\theta }\left(h_{i}\right)\right)\text{.}$$

The divergences $\text{KL}\left(q\left(h_{i}\right)∥p_{\theta }\left(h_{i}\text{|}x_{i}\right)\right)$ in Eq. (5) are always non-negative. Thus, for any joint distribution $q={\left\{q_{i}\right\}}_{i=1\cdot n}$, the function $L\left(q,\;X,\;\theta \right)$ provides a valid lower-bound to $L\left(X,\;\theta \right)$ (see Fig. 2), called the Evidence Lower Bound (ELBO):

$$\forall q,\;\forall \theta ,\;L\left(q,\;X,\;\theta \right)⩽L\left(X,\;\theta \right)\text{.}$$

The lower-bounds $L\left(q^{\left(t\right)},\;X,\;\theta \right)$ are proxies for $L\left(X,\;\theta \right)$, that - in contrast to $L\left(X,\;\theta \right)$ - can be computed and maximized in θ.

#### Inference Methods Based on an ELBO.

While the ELBO holds for any q, some choices are more judicious than others. In fact, the goal is to select an optimal q, such that the gap between $L\left(q,\;X,\;\theta \right)⩽L\left(X,\;\theta \right)$ is small: this will insure that the maximization of $L\left(q,\;X,\;\theta \right)$ with respect to θ yields estimates $\theta^{*}$ that are also appropriate (and close to the true optimum $\theta^{\text{true}}$) for maximizing $L\left(X,\;\theta \right)$ — see Fig. 2 (right). Inference methods in cryo-EM subsequently differ in the choices of the distributions $q_{i}^{\left(t\right)}$ for each i and at each iteration t, thereby yielding different lower bounds $L\left(q,\;X,\;\theta \right)$:

- **Using the posteriors given current parameters (EM algorithm):** Computing the posteriors $p_{\theta }\left(h_{i}\text{|}x_{i}\right)$ using the current estimated value $\theta^{\left(t\right)}$ of θ allows choosing $q_{i}^{\left(t\right)}\left(h_{i}\right)=p_{\theta^{\left(t\right)}}\left(h_{i}\text{|}x_{i}\right)$ for each i at iteration t — see Figure on the posterior distributions in the supplementary material. The inequality:
$$L_{i}\left(p_{\theta^{\left(t\right)}}\left(h_{i}\text{|}x_{i}\right),\;X,\;\theta \right)⩽L_{i}\left(X,\;\theta \right),$$
  becomes an equality for $\theta^{\left(t\right)}=\theta$. This makes the lower-bound $L\left(q,\;X,\;\theta \right)$ tangent to $L\left(X,\;\theta \right)$ at $\theta =\theta^{\left(t\right)}$: progressively maximizing $L\left(q,\;X,\;\theta \right)$ with respect to θ will induce convergence to a local maximum of $L\left(X,\;\theta \right)$ in θ, as seen in Fig. 2 (left). This is the strategy adopted by Expectation–Maximization (EM) algorithm (more details in the supplementary material). The EM is an iterative algorithm which consists of two steps. In the first step (called the expectation step), given current parameters values $\theta^{\left(t\right)}$, we compute the posterior $q_{i}^{\left(t\right)}\left(h_{i}\right)=p_{\theta^{\left(t\right)}}\left(h_{i}\text{|}x_{i}\right)$ to plug into our ELBO. In the second (the maximization step), $\theta^{\left(t+1\right)}$ is taken to be the value of θ that maximizes the ELBO. This sequence of two steps is usually repeated until convergence. As explained in the supplementary material, while the EM algorithm does not have any convergence guarantees, it nonetheless guarantees to increase the likehood at each step.
- **Approximating the posteriors given current parameters (Variational EM algorithm):** In certain cases, the choice of $q_{i}^{\left(t\right)}\left(h_{i}\right)$ as the posterior $p_{\theta^{\left(t\right)}}\left(h_{i}\text{|}x_{i}\right)$ is neither computationally attractive nor feasible. In this case, we might prefer approximating each posterior by finding its “best approximation” $q_{i}^{*}$ within a family of functions called variational family Q. Cryo-EM reconstruction methods consider two choices that include approximating the posteriors by *(i) their “mode”, i.e. the value ${\hat{h}}_{i}$ of $h_{i}$ that maximizes $p_{{\tilde{\theta }}^{\left(t\right)}}\left(x,\;h\right)$*. In this case, each $q_{i}$ effectively becomes a Dirac distribution at ${\hat{h}}_{i}$; or *(ii) or a general distribution $q_{i}$ within a family Q*: $q_{i}$ is for example a Gaussian distribution – see Figure presenting the posterior distributions in the supplementary material.

#### Exact or Approximate Posteriors: Pros, Cons, Discussion.

The EM algorithm, that uses exact posteriors, holds several advantages: it is simple and stable, since all updates can only improve the observed log-likelihood. However, it is also potentially slow: the rate of convergence is known to be linear with rate proportional to the fraction of information about θ in $L\left(\theta ,\;X\right)$ (Dempster et al., 1977). Variational EM algorithms can be faster; yet they potentially loose in accuracy as their ELBOs do not provide tight lower-bounds to the log-likehood $L\left(X,\;\theta \right)$ (Fig. 2, right). As a result, we do not have any guarantee that they converge to an (even local) maximum of $L\left(X,\;\theta \right)$.

### 3.2. Introducing amortized inference

While potentially more computationally attractive than the original EM algorithm, Variational EM requires solving n optimization problems to find an approximate posterior $q_{i}$ for each image i in 1,…, n. This is computationally expensive, as the number of $q_{i}$ to estimate increases as the number of images n increases. Consequently, recent methods have resorted to using an additional approximation called Amortized Inference (AI), which collapses the n optimizations problems into one. Instead of finding the best corresponding $q_{i}^{*}$ for each i, AI optimizes the parameters ξ of a function ${\text{Enc}}_{\xi }$ that predicts the parameters of the distribution $q_{i}^{*}\left(h_{i}\right)$ when given $x_{i}$ as input, i.e.: $\left.{\text{Enc}}_{\xi }\left(x_{i}\right)≃\left(E\left[h_{i}\right],\;\text{Var}\left[h_{i}\right]\right)\right)$, where, in this example, the variational family Q is chosen to be the set of Gaussian distributions. In other words,instead of solving n separate problems, Amortized Inference predicts the parameters of the posterior of image i using the observed image as input. The function ${\text{Enc}}_{\xi }$ is traditionally called an encoder. More details — including a description of updates performed in AI — can be found in the supplementary material.

#### Implementation of Amortized Inference with Variational Autoencoders

In cryo-EM reconstruction, amortized inference is deployed in the context of variational autoencoders, denoted VAEs. VAEs are deep architectures that model the parameters of the variational family ${\text{Enc}}_{\xi }$ described above as a neural network with weights ξ — therefore leveraging the expressivity of this class of functions to get an optimal (amortized) variational approximation. The entire VAE pipeline thus consists of two steps: an encoder, which is simply a neural network with weights ξ corresponding to the function ${\text{Enc}}_{\xi }$ described above, and a decoder, which allows to create “mock samples” that will then be compared with the observed ones based the generative model with parameter θ chosen in Section 2. Here, the encoder is either a variant of a convolutional neural network (if the input $x_{i}$ is in the image domain), or variants of fully connected networks (if the input $x_{i}$ is in the Fourier domain). The decoder is almost entirely dictated by the process described in Eq. 1 and goes beyond the conventional fully connected networks or convolutional neural networks used in image processing.

**Fig. 3.**
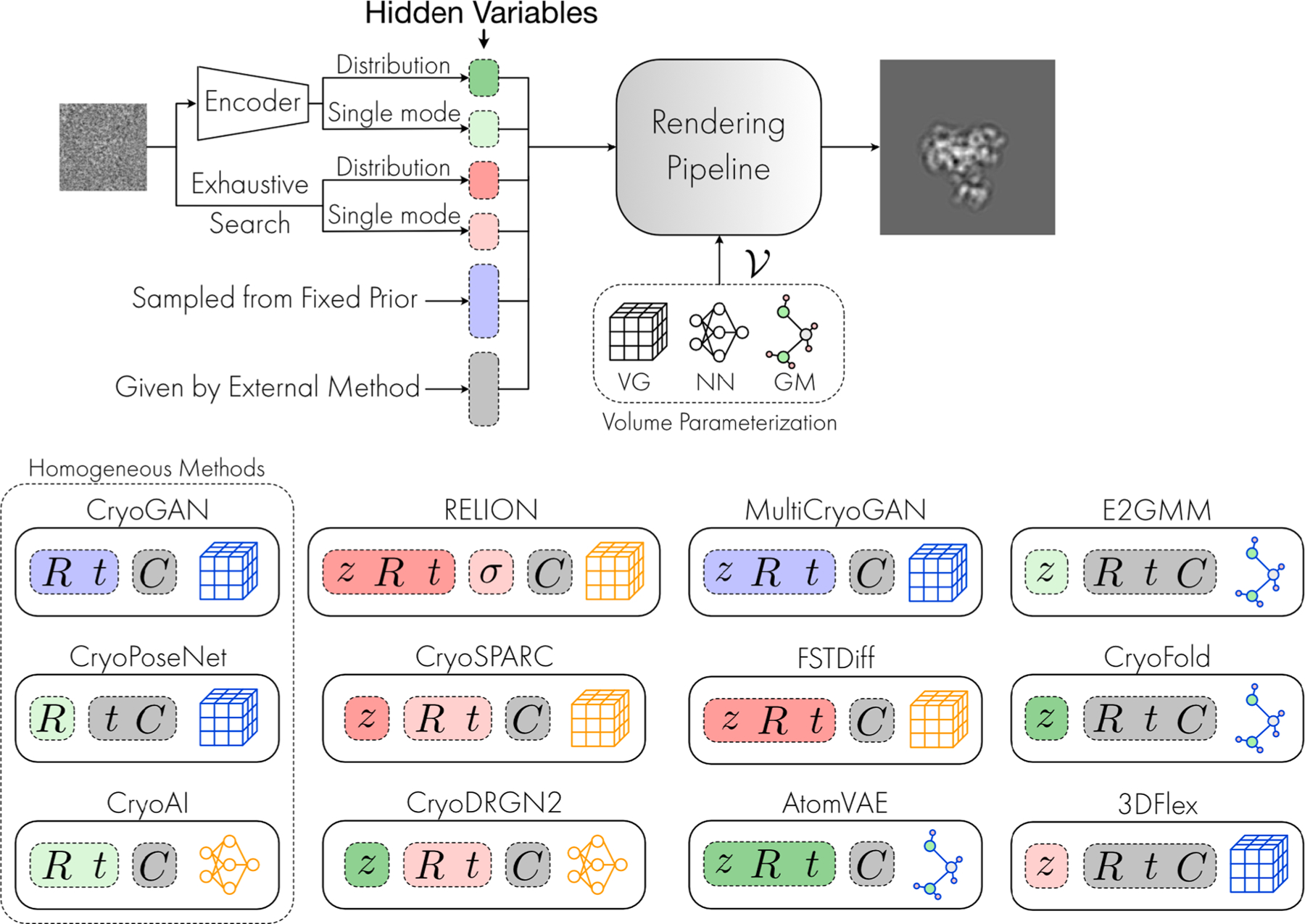
Comparison of generative reconstruction methods. *Volume Representation (see* Section 2*):* methods parameterizing volumes with a discretized domain and explicit parameterization are shown with a voxel grid (VG) pictogram; methods parameterizing volumes with a continuous domain and implicit parameterization are shown with either a neural network (NN) or a Gaussian mixture (GM) pictogram; the pictogram is outlined in blue for volumes represented in image space and in orange for volumes represented in Fourier space. *Inference (see*Section 3*):* Each method is shown with the hidden variables of its generative model, *i.e.*, which are some combination of 3D rotation R, 2D translation t, contrast transfer function C, standard deviation of measurement noise σ, and conformation variable z. In each method: hidden variables assumed known are shown in gray; hidden variables sampled from a fixed prior, in generative adversarial network (GANs) architectures, are shown in blue; hidden variables computed with the expectation–maximization algorithm (EM) are shown in red: dark red for a variational EM that produces an approximation of their posterior distribution, and light red for a modal EM that produces a single mode; hidden variables computed with amortized inference through an encoder are shown in green: dark green for a variational encoder that predicts the parameters of an approximation of their posterior distribution, light green for a regular encoder that predicts a single mode *Remark:* CryoDRGN (Zhong et al., 2021) is similar to cryoDRGN2 (Zhong et al., 2021), except rotations and translations are given by an upstream homogeneous reconstruction. CryoVAEGAN (Miolane et al., 2019) does not explicitly store a representation of the volume and therefore does not appear in the figure.

The learnable parameters ξ and θ of the encoder and the decoder are fitted through stochastic gradient descent via backpropagation of the ELBO through the neural network. Compared to traditional cryo-EM reconstruction methods leveraging the EM algorithm, variational Autoencoders can be interpreted as extending the E-step (encoder) and the M-step (decoder) of the EM algorithm. The VAE architectures of cryo-EM reconstruction methods using amortized inference are given in Fig. 3. In this review, we have also included for comparison purposes a non-variational version of this procedure: the autodecoder from 3DFlex (Punjani and Fleet, 2021). Here, the authors consider the hidden variables as non-random variables, but add a fix amount of gaussian noise to regularise the embeddings. While the final loss is therefore adapted, this is essentially a VAE where the variance is fixed, while only the mean is learned.

#### Amortized Inference: Pros, Cons, Discussion

Amortized inference is faster than its non-amortized counterparts, but adds an additional error (called the amortization error). We observe that several methods use amortized inference, but often to estimate one hidden variable: e.g. only the rotation R or only the conformation variable z. Fig. 3 classifies the reconstruction methods by the type of inference chosen for each variable within $h_{i}=\left(R_{i},\;t_{i},\;{\text{PSF}}_{i},\;z_{i}\right)$ and indeed, we note that this choice does not have to be consistent across all hidden variables. Many methods “mix and match” inference techniques, using for example a variational EM for the hidden rotation variable $R_{i}$ and a VAE for the conformation variable $z_{i}$. Moreover, it becomes apparent from Fig. 3 that (variational) autoencoders are the most common type of approaches implemented for cryo-EM reconstruction.

While deep generative methods for cryo-EM volume reconstruction can be unified with the framework described above (as well as with traditional Expectation Maximization approaches), we observe that each of them has its own specificities or “implementation tricks”. They differ, for example, in their choice of variational family, or loss function that adapts the ELBO to facilitate convergence of the optimization procedure, see supplementary material. These testify to the difficulties encountered in training these algorithms in the context of cryo-EM images with low signal–noise ratios.

## 4. Discussion

Given the wide number of options to reconstruct molecular volumes from cryo-EM images, it is natural to ask: *which reconstruction method is in fact the most promising?* In this last section, we focus on the need for establishing a set of metrics and benchmark tasks that can be used to quantitatively compare the performance of these methods. Starting with a review of the tools currently available, the first take-away of this section is the urgent need for new metrics and benchmarks. The evaluation of these methods’ performance is currently difficult and inherently limited. We nonetheless highlight, as a second take-away, promising features in current developments, which, in our opinion, these developments pave the way for future improvements in cryo-EM reconstruction.

### 4.1. Assessing reconstruction performances: need for new metrics

Performance metrics can be categorized in two classes: (a) those that assess a method’s ability to provide good **spatial resolution** (i.e. distinguishing different atoms), and, in the case of heterogeneous methods, (b) those that assess a method’s ability to provide good **conformation resolution** (i.e. distinguishing different conformations).

#### 4.1.1. Assessing spatial resolution

##### Resolution of discretized reconstructions (3D maps).

When the reconstructed volume is parametrized as an explicit 3D map, the most widespread measure used to evaluate its spatial resolution is the Fourier Shell Correlation (FSC) (Harauz and Heel, 1986). As described by Singer et al. (2020), this quantity measures the correlation over a 3D shell between two reconstructed volumes:

(7) $$FSC_{k}\left(U,\;V\right)=\frac{\sum_{s\in S_{k}}U_{s}V_{s}^{*}}{\sqrt{\sum_{s\in S_{k}}{\left|U_{s}\right|}^{2}\sum_{s\in S_{k}}{\left|V_{s}\right|}^{2}}}\text{.}$$

Here, $S_{k}$ is the set of Fourier voxels in a spherical shell at distance k from the origin, and U and V are the Fourier transforms of the 3D volumes that we compare. Typically, U and V correspond to two independent reconstructions on separate halves of the dataset, in which case, the criterion for a method to be deemed to perform well is for the two reconstructed volumes to be similar. The method’s resolution is then defined as the highest resolution for which U and V agree “enough”. This is precisely what the FSC (Eq. 7) captures: the FSC is close to 1 when the two maps are close. This is usually the case for small k, as low-frequency signal is strong, but the FSC generally decays to zero as the signal-to-noise decreases. The result is often plotted as a curve, with axis x=k. The resolution of the reconstruction corresponds to the maximum value of k such that $FSC_{k}⩾0.143$ — a criterion chosen to match resolution criteria used in X-ray crystallography (Marin and Schatz, 2005). For synthetic datasets where a ground-truth volume is available, the FSC is measured between the reconstruction and the ground-truth; in which case the resolution criterion correspond to the maximum value of k such that $FSC_{k}⩾0.5$.

##### Resolution of continuous reconstructions.

Methods that represent the volume as a continuous field are relatively new, and it might be worth reassessing appropriate metrics for evaluating spatial accuracy in this case.

- **Implicit Parametrizations** While interpolation between image pixels and map voxels is necessary in the discrete case, both for projection and for backprojection, implicit representations of the volume (e.g., through an neural network) enable sampling without interpolation during training. It would be interesting to investigate whether this provides a benefit in terms of reconstruction quality. We do not expect implicit representations to suddenly *unlock* information, since the information content is determined by the discrete nature of the images and their pixel size, but they might provide new actionable ways to implement prior informations about the volume, such as smoothness and stereochemistry, that would result in reconstructions of higher quality.
- **Atomic Parametrizations** Parametrizations of the volume with atomic models represents an opportunity to revisit the notion of spatial resolution. The traditional measure of similarity between two atomic models that only differ in the cartesian coordinates U and V of their N constituting atoms (using a consistent orientation of the molecule for U and V) is the Root Mean Square Deviation (RMSD). This quantity is defined as:
(8) $$RMSD\left(U,\;V\right)=\sqrt{\frac{1}{N}\sum_{i=1}^{N}{\left|U_{i}-V_{i}\right|}^{2}},$$
  However, it was soon recognized that this metric had a very narrow range for interpretability (Kufareva and Abagyan, 2011): it is a global measure of similarity, which is not suited to capture the local — but meaningful— changes in protein structure. To complicate the matter, measuring the RMSD between two atomic models assumes that they are both in the same reference frame, which might not always be defined. To improve the sensitivity of the metric, atomic models are routinely reduced to features with desired properties, e.g. vectors of internal coordinates independent of the reference frames. For example, reducing the atomic model to its backbone dihedral angles or to a list of atomic contacts has been shown to yield better clustering of conformations (Scherer et al., 2019). The development of new established metrics to evaluate these models is thus an important avenue of development.

#### 4.1.2. Assessing conformation resolution: an Ill-Defined Problem

While not flawless, the evaluation of spatial resolution is a relatively well-characterized task. By contrast, evaluating conformation heterogeneity is a more ambiguous problem. To evaluate the quality of the reconstructions allowing continuous heterogeneity, methods such as 3DFlex (Punjani and Fleet, 2021) or CryoDRGN (Zhong et al., 2019) perform a post hoc analysis of the recovered latent space, showing the flexible deformation that are induced by sweeping through the space of possible zs and visually inspecting the corresponding deformations. However, proper objective and quantitative measures of conformation heterogeneity remain to be established: there currently exists no standardized measure or gold-standard task to evaluate how well a method is able to capture it.

We could design a new metric, inspired by the high-level idea of the FSC. Using two halves of the dataset to infer two independent continuous distributions of conformations, we evaluate whether the distributions agree using a metric such as the Wasserstein distance - modulo change of coordinate system for the conformation variable z. In the case where a ground-truth conformation is available for each image (e.g. in simulations), the inferred distribution could be compared to the true distribution. In the case of methods able to generate one 3D volume for each image in the dataset, one could consider a hierarchical clustering approach where depth in the hierarchy tree corresponds to the conformational resolution. In more concrete terms, for all resolution k, the FSC between each volume pairs would be measured and the resulting distance matrix used for clustering. Data points that fall within the same clusters would be indistinguishable at k while images that would fall in different clusters would correspond to conformations that differ by at least k. The development of such metrics is key to make sustainable advances in next-generation cryo-EM reconstructions.

### 4.2. Quantitative comparison of performances: lack of common benchmarks

Beyond the need for new performance metrics that are better adapted to new advancements in the field, it is most certainly the lack of established benchmarks that, to this day, make reconstruction methods very hard to compare. Such benchmarks are dramatically needed, as we cannot rely on statistical theory since the convergence properties of estimations relying on (amortized) variational inference are not completely characterized. In fact, the quantitative assessment of the methods’ relative performance has yet to overcome three main hurdles:

- **Lack of benchmark datasets:** Current methods are developed and tested on a wide range of synthetic and experimental datasets that differ in the nature of the biomolecule being imaged, the dataset size, image size and associated resolution — with very little overlap across methods - see Table summarizing existing experiments in the supplementary material. There is unfortunately no MNIST (Deng, 2012) or Imagenet (Deng et al., 2009) for cryo-EM. Most methods resort to evaluating their performance on synthetic data, yet no cryo-EM simulator acts as a standard to generate simulated images in a unified way. Synthetic datasets vary in the realism of the image formation model used for simulation, *e.g.* in the noise model, the signal-to-noise ratio or the distribution of nuisance variables (e.g. poses). Subsequent experiments are typically performed on real “in house” data — but there too, the important diversity within the characteristics of these evaluation datasets therefore makes the comparison of these methods a strenuous task.
- **Lack of benchmarking procedures:** Reconstruction methods vary in the complexity of the task that they set out to accomplish, assuming more or less nuisance variables (such as poses or PSF) to be known – see Fig. 3. This makes it difficult to compare methods on a fair ground. We need to establish modular benchmarking procedure that would enforce a fair comparison of reconstruction performances, eg, testing the recovery of the pose, volume or conformations, with other nuisance variables being known and fixed.
- **Lack of benchmark codebase and infrastructure:** Finally, reconstructions methods are not necessarily publicly accessible, are implemented across different programming languages, and/or are tested on different software or hardware. Creating a codebase that re-implements these methods for a proper evaluation using a unified infrastructure would unfortunately represent a gigantic implementation effort. Currently, this lack of codebase poses a significant hurdle in the accessibility and comparison of the methods: it is currently impossible to disentangle the effect of their proposed statistical learning problem, their programming language, implementation tricks, or software infrastructure.

### 4.3. Qualitative comparison of performances

Despite the hurdles associated with performing quantitative comparisons, we propose a qualitative evaluation of the different methods based on both published results and our personal experience. This allows us to highlight promising directions — to the least, in the authors’ opinion — for further developments.

#### 4.3.1. Accuracy

Despite encouraging accuracy results, some methods seem to face considerable challenges when applied to real cryo-EM images, as they have not been properly vetted and stress-tested in experimental conditions (Ullrich et al., 2019; Zhong et al., 2021; Rosenbaum et al., 2021; Nashed et al., 2021) – see Table summarizing the experiments in the supplementary material. We consider the lack of results on experimental data as a proxy for a limited applicability in the context of real signal–noise ratios regimes. In order to be adopted by cryo-EM practitionners, these methods will need to overcome the signal–noise regime and demonstrate the accuracy reported in the papers on a larger set of (benchmark) datasets.

Despite the difficulty of the task and lack of standardized benchmarks, recent developments in deep generative modeling have shown impressive promise in overcoming the current computational and accuracy bottlenecks in all three following directions:

- **Poses:** Poses are important nuisance variables that have the potential to damage the reconstruction, if incorrectly predicted. Accuracy of the predicted rotation is measured on synthetic datasets with a mean/median square error (MSE) against the corresponding ground-truth. Historically, preference was given to methods that did not use amortized inference for the rotation estimation (e.g. CryoSPARC (Punjani et al., 2017) or CryoDRGN (Zhong et al., 2019)), as they outperformed their amortized counterparts predicting rotations with an encoder (e.g. CryoPoseNet (Nashed et al., 2021) and CryoAI (Levy et al., 2022)): AtomVAE (Rosenbaum et al., 2021) was for instance one of the only methods using amortized inference for the recovery of the poses, and reported difficulties in the joint training of poses and conformation — highlighting the difficulty of accurate amortized inference in this setting. However, the accuracy gap between methods is closing: CryoAI (Levy et al., 2022) now showcases an rotation accuracy at the same order of magnitude compared to CryoSPARC (Punjani et al., 2017) and CryoDRGN (Zhong et al., 2019) on a real dataset. This was facilitated by the theoretical insights drawn from CryoAI (Levy et al., 2022), who show that amortized inference techniques tend to get stuck in local minima where the predicted molecule contains unwanted planar symmetries due to their projections on a 2D surface. The solution that they propose to alleviate this problem is to use the symmetrized loss:
$$\ell_{\text{sym}}=\sum_{i}\text{min}\left\{{\left\|X_{i}-{\text{PSF}}_{i}*\left(t_{i}\circ {\text{Π}}_{2D}\circ R_{i}\right)\left(V^{\left(i\right)}\right)\right\|}^{2},\;{\left\|R_{\pi }\left(X_{i}\right)-PSF_{i}*\left(t_{i}\circ {\text{Π}}_{2D}\circ R_{i}\right)\left(R_{\pi }\left(V^{\left(i\right)}\right)\right)\right\|}^{2}\right\}\text{.}$$
  where $R_{\pi }$ is a rotation with angle π. This has recently opened the door to significant gains in accuracy in the prediction of the poses, allowing for the first time pose estimation to be done through amortized inference. We anticipate that it is through such developments and theoretical insights that reconstruction algorithms will be able to fully leverage amortized inference for rotation prediction, hereby providing significant speed-ups.
- **Volumes:** Methods based on (pseudo-) atomic volume parametrizations - E2GMM (Chen and Ludtke, 2021), CryoFold (Zhong et al., 2021) and AtomVAE (Rosenbaum et al., 2021) - do not compare themselves to their counterparts, probably due to the fact that they were published concurrently in 2021 and/or do not use the same definition of “pseudo-atoms” that are respectively: means of 3D Gaussian distributions, residues or actual atoms. As a consequence, we do not comment on them. For methods generating 3D maps, it has been reported by Punjani and Fleet (2021) that amortized inference can translate into resolution loss. Yet, recent methods such as CryoDRGN (Zhong et al., 2019) and CryoAI (Levy et al., 2022) publish examples of reconstructed volumes as 3D maps whose resolution is visually comparable to the ones obtained by CryoSPARC (Punjani et al., 2017), on downsampled imaged. Our opinion is that amortized methods can reach near-atomic resolution reconstructions, but this has not been demonstrated yet. If so, we expect them to replace traditional reconstruction pipelines in the long run, since they offer the promise to be significantly faster and to tackle much larger datasets - a desired feature to enable sufficient sampling of the conformational landscape.
- **Conformations:** Methods based on pseudo-atomic volumes parametrizations do not provide examples of conformation trajectories that allow us to compare them. For methods generating 3D maps, CryoDRGN (Zhong et al., 2019) and 3DFlex (Punjani and Fleet, 2021) seem to be some of the most promising approaches, as they seem to allow greater resolutions in the recovered trajectories, based on our personal visual assessment of the examples of conformation trajectories shown in the corresponding papers. This remains to be confirmed by a quantitative assessment over a larger number of conformation trajectories.

#### 4.3.2. Reproducibility

Adoption of these methods by practitioners will require their reproducibility, or robustness to different initializations, implementation tricks or choice of hyperparameters:

- **Initialization:** The non-convex nature of the problem puts it at very high-risk of being non robust and sensitive to initialization — this is a phenomenon sometimes referred to as “Einstein from noise” (Henderson, 2013), also described in Singer et al. (2020). Luckily, most of the current methods show encouraging signs of robustness to perturbations. In our experience, CryoDRGN (Zhong et al., 2019) seems consistent for different random initializations of the neural model when fixing the poses: the conformation space does not seem to be vastly affected. This robustness can however be challenged by extremely low signal and/or heterogeneous datasets, in which case certain conformations can go missing.
- **Tricks:** The inference methods presented often make use of additional implementation tricks (e.g. warm-starting with a known conformation), and specific regularization schemes: e.g. AtomVAE (Rosenbaum et al., 2021) suggests starting with an initial phase of pose-only training, which, once realized, ensures that the further joint learning of poses and volume is successful. Both AtomVAE (Rosenbaum et al., 2021) and CryoFold (Zhong et al., 2021) regularize the recovered structure by penalizing bond lengths, but the impact of the regularization yet remains to be properly characterized, and in particular, its potential to frustrate the optimization landscape. The importance of tricks and regularizations, and combinations thereof, is still ill-understood and would require an in-depth analysis, as it hints towards a difficult optimization landscape for this method, and its sensitivity to initial conditions.
- **Hyperparameters:** Choosing hyperparameters such as the dimension of the latent space in algorithms such as CryoDRGN (Zhong et al., 2019) induces more or less regularization: too small and it regularizes the model too much; too large leads to underfitting of the 3D model. CryoDRGN (Zhong et al., 2019) usually sets it to d=8, but, given how heterogeneity arises, this is necessarily molecule dependent. The field will need — to the least— rule-of-thumb guidelines on how to choose these hyperparameters if these methods are to be adopted by practitioners.

The robustness of these new methods needs to be confirmed on a wider set of datasets, including datasets with high levels of noise. The fact that they rely on user-defined implementation tricks and hyperparameters might not be an obstacle to their adoption, as conventional methods such as RELION (Scheres, 2012) or CryoSPARC (Punjani et al., 2017) also do.

#### 4.3.3. Efficiency

Our last axis of comparison is computational efficiency: both in time and memory requirements. First, if we take the size of the datasets used in experiments as a proxy for efficiency, then 3DFlex (Punjani and Fleet, 2021), CryoAI (Levy et al., 2022), E2GMM (Chen and Ludtke, 2021), and CryoDRGN (Zhong et al., 2019) seem to be able to process remarkable amounts of information. Additionally, we offer our own practical experience by-way of rule of thumb. With datasets typically of more than 100 GB, training times can take up to 10 h (including the required pre-processing steps) for methods like RELION (Scheres, 2012) — that is, for a run that has little hyperparameter tuning compared to alternative deep learning methods. Newer methods like CryoDRGN (Zhong et al., 2019) hold great promise in terms of reconstruction: however, such sophisticated methods can further benefit from gains in efficiency, both from the computational side and in terms of memory requirements. Efficient updates of a model’s parameters can thus be seen as a current computational bottleneck and offers an interesting avenue for future research.

## 5. Conclusion

This review provides a critical comparison of recent cryo-EM reconstruction methods that are based on deep generative modeling, focusing on explaining their relative advantages or drawbacks. We have unified, compared and contrasted existing methods through their parametrization of the volume, as well as through the optimization procedure chosen to recover this volume and associated hidden variables. While the use of amortized inference is crucial to make inference tractable in this high-data, high-dimensional setting, there seems to be much room for improvement and research on methods allowing both faster and better inference. On a practical side, we note that recent methods suffer from a lack of benchmarks which severely impedes their comparison and development. From our practical experience, beyond a necessity for benchmark datasets, we also highlight a severe need for the development of a diagnostic toolbox tailored to the analysis of cryo-EM data. Current methods rely on a set of choices and hyperparameters that raise a number of questions for the practitioner: have I chosen my hyperparameters adequately? Is this choice going to impact the accuracy of the recovery? Is there any physical or biological meaning or interpretation in the distance between the latent space of conformation variables? How does error on pose or PSF affect the rest of the volume recovery process? There is therefore a pressing need for more in-depth and systematic quantitative comparison of these methods.

## Supplementary Material

supplementary

## Abbreviations:

cryo-EM: cryo-electron microscopy
PSF: point spread function
CTF: contrast transfer function
ELBO: Evidence Lower Bound
EM: Expectation Maximization
FSC: Fourier Shell Correlation
