# Supplementary material for: Deep generative modeling for volume reconstruction in cryo-electron microscopy
Source: J Struct Biol. Author manuscript; Available in PMC 2023 Aug 18. (PMC10437207; doi:10.1016/j.jsb.2022.107920)
Supplement: supplementary [file NIHMS1918890-supplement-supplementary.pdf]

Supplementary Materials: Deep Generative Modeling for Volume Reconstruction in Cryo-Electron Microscopy

Claire Donnat[1+], Axel Levy[2,3], Frédéric Poitevin[3], Ellen Zhong[4], and Nina Miolane[5*+]

[1]University of Chicago, Department of Statistics, Chicago, Illinois, USA
[2]Stanford University, Department of Electrical Engineering, Stanford, CA, USA
[3]LCLS, SLAC National Accelerator Laboratory, Menlo Park, CA, USA
[4]Massachusetts Institute of Technology, Computer Science and Artificial Intelligence Lab, Boston, MA, USA
[5]University of California Santa Barbara, Department of Electrical & Computer Engineering, Santa Barbara, CA, USA
[*]ninamiolane@ucsb.edu
[+]these authors contributed equally to this work

ABSTRACT

This is the supplementary materials to the critical review: "Deep Generative Modeling for Volume Reconstruction in Cryo-Electron Microscopy".

Appendix A: Ab-initio vs Refinement Reconstructions

The main text of the present critical review establishes how cryo-EM methods target either homogenenous or heterogeneous reconstruction, and refined these categories by drawing a distinction between methods that are reference-based, and those that are reference-free — that is, those that do not try to parametrize heterogeneity as "small" deviations from a reference shape. Here, we introduce an additional nuance between these methods, and differentiate between methods that operate *ab-initio* (ie. from scratch) , or *by refinement* (ie, "warm-starting " the algorithm by using an existing and external volume estimate, such as for example a low resolution estimate). While, for the sake of clarity and conciseness, we did not elaborate on this distinction in the main text, the latter introduces an other (independent) axis of variation in our comparison of existing methods. In fact, methods can target reference-free heterogeneous reconstruction whilst warm-starting their algorithm close to a known solution (as is done in the heterogeneous version of RELION[29]), or vice versa, prefer to opt for reference-based heterogeneity, with no prior knowledge of what the reference should look like (e.g. 3DFlex[32]).

This distinction can be understood as a way of infusing further knowledge into the recovery. *From a statistical and compute science perspective*, choosing to operate by refinement depends on whether or not we have a reasonable prior for the shape (refinement), or if we'd rather not bias the algorithm with external inputs (ab initio). In this high-dimensional (and highly non-convex) setting, imposing a specific starting point for the algorithm is in fact likely to introduce an additional bias to the solution — thereby potentially allowing it to recover more easily interesting structures. Conversely, in the absence of good guesses, ab initio methods prefer to use a random initialization. *From a biological perspective*, this boils down to how much we trust potential external reconstruction methods, and we think that they could be instrumental in recovering the structure.

Ab-initio vs refinement: Pros, Cons, Discussions. Figure 1 summarizes the methods performing ab-initio reconstruction versus refinement. While "bias-free", ab-initio methods can be challenging to fit: due to the highly non-convex, non-linear nature of the problem, these methods are at higher risk of inconsistencies, especially when targeting an heterogeneous reconstruction. In fact, solutions initialised at various random points might not necessarily converge to same point, and/or get stuck in local minima. By contrast, warm-starting the problem can yield faster, and more consistent convergence towards the solution. Warm-starting the solution might also increase consistency across recovered conformation, thereby preventing aberrations. Homogeneous reconstruction approaches can also benefit from warm-starting strategies: in RELION[13] for instance, the hypothesised template is converted into Fourier coefficients, that are used in the initialisation part of the inference process. Refinement for homogeneous reconstruction holds several advantages, especially if the homogeneous reconstruction is either slow or very sensitive to initialization.

Yet, refinement also holds several disadavantages. This approach requires a two-step procedure and another fragmentation of the estimation pipeline, as it relies on a prior estimation of an initial "blunt" value by another pipeline. Moreover, as highlighted

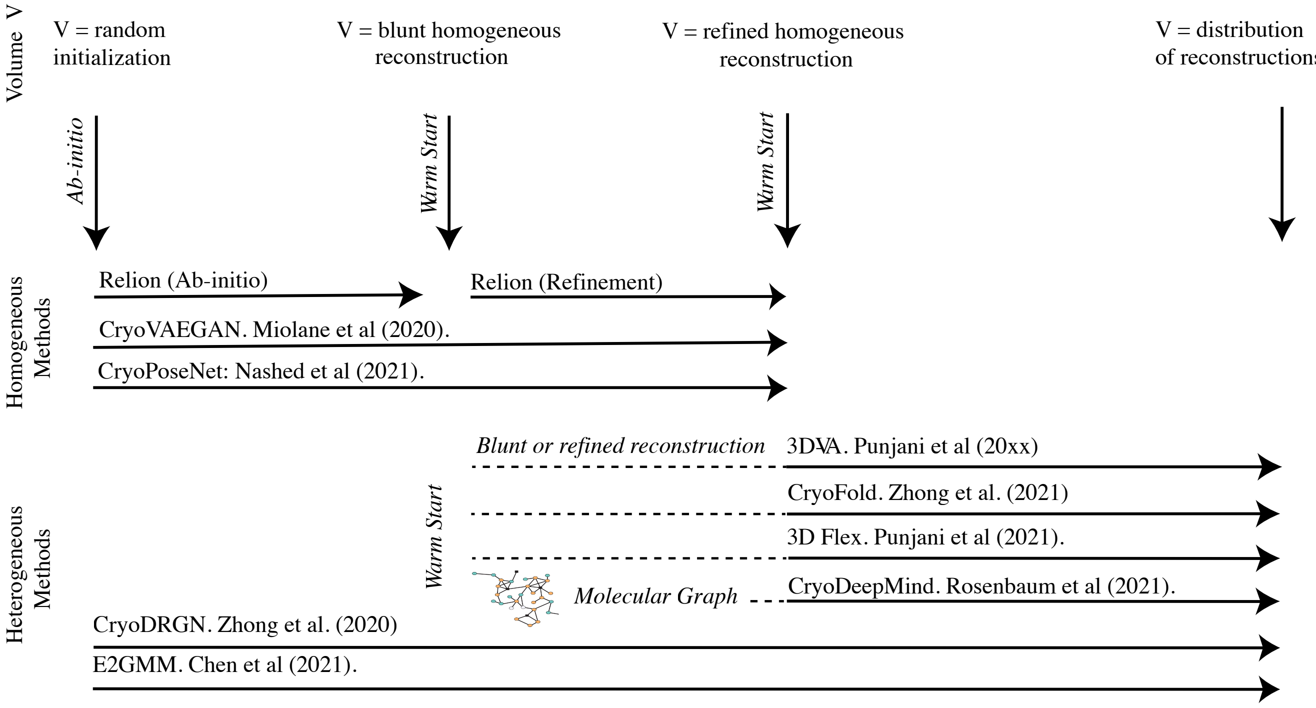

Figure 1. Works differentiated by the objective of the reconstruction (homogeneous/heterogeneous), and its initialization (ab-initio/warm-start).

in the review of Bendory et al[20], it also exposes this solution to model bias: the structure recovered will be biased towards our initial prior — potentially hindering our ability to detect flaws in this original template or discovering new conformations. Finally, while the literature on the sensitivity of the methods to an erroneous warm-starting is also nonexistent, the impact of a wrong initialization could also either severely hinder the performance of the algorithm, or push the algorithm to hallucinate nonexistent solutions (allowing us to recover "Einstein from noise"[47]— see discussion in the main text).

Appendix B: Differentiability of the Generative Models

An alternative (and often simpler) generative model can be formulated via the Fourier equivalent of the Equation representing the generative model in the main text.

Fourier-Slice Theorem. In Fourier Space, the convolution $*$ of the projected 2D volume by the PSF conveniently becomes an element-wise matrix multiplication $\odot$ between the 2D Fourier transform of the projected image and that of the PSF. The latter is better known as the Contrast Transfer Function (CTF), with $\text{CTF}_i = \mathcal{F}(\text{PSF}_i)$, so that:

$$\tilde{X}_i = \text{CTF}_i \odot (P_i^{(R_i,t_i)} \circ \tilde{V}^{(i)}) + N_i, \qquad \text{with} \quad i = 1 \cdots n. \tag{1}$$

Here, $\tilde{X}_i$ is the 2D-Fourier transform of the observed image X_i ($\tilde{X}_i = \mathcal{F}(X_i)$), and $\tilde{V}^{(i)}$ is the 3D Fourier transform of the molecule (i.e, the volume) $V^{(i)}$, with $\tilde{V}_i = \mathcal{F}(V_i)$. In Equation (1), the operator $P_i^{(R_i,t_i)}$ first extracts out of $\tilde{V}^{(i)}$ a slice oriented by the rotation R_i and then applies a phase shift in Fourier space accounting for the 2D translation t_i. Finally, N_i represents the noise. We note that using the operator P_i circumvents the 3D-to-2D projection step and is therefore more computationally efficient.

Efficiency and differentiability of the different generative models The methods described in this review leverage the ability to do inference with gradient-based optimization techniques. This requires the generative models to be differentiable — that is, to allow the differentiation of the loss associated with the output (an image) with respect to the parameters of the model through backpropagation. As explained in the main text, the volume V (or $\tilde{V}$ in Fourier space) can always be seen as a function from $\mathbb{R}^3$ to $\mathbb{R}$ (or $\mathbb{C}$). The parameters defining this function depend on the choice of *parametrization*, as summarized in Table 1. To make sure that the model is differentiable, we need to ensure that all the operations required in the forward model presented in image space in the main text are differentiable. Table 1 summarizes the operations required to "rotate", "translate" and "project" the volume V and obtain the observed image. Those operations are:

- multiplication with a matrix $R \in \mathbb{R}^{3\times 3}$,

- addition of a vector $\mathbf{t} \in \mathbb{R}^3$,
- application of an interpolation kernel,
- summation (if the volume is in image space),
- and analytical integration.

All these operations, if they involve the “differentiable parameters” can be differentiated through by backpropagation. As shown in the equations of the image formation model (either in image space or in Fourier space) the last step of the generative model is the application of the PSF (resp. CTF) which is done with a convolution (resp. element-wise multiplication) with the projected volume. This last operation is also differentiable, which in turn ensures that the whole generative model, transforming a volume V into an image (potentially in Fourier space), is differentiable.

	**Differentiable Parameters**	**Domain**	**Rotation Translation**	**Projection (in image space)**
Voxel Grid	$V(x)$ for $x \in \{1,\dots,D\}^3$	$V : \{1,\dots,D\}^3$ With interpolation kernel, $\hat{V} : \mathbb{R}^3$, see[39]	$\hat{x} = R^{-1}x - \mathbf{t}$	$\sum_x \hat{V}(\hat{x})$ $\hat{V} : \mathbb{R}^3 \to \mathbb{R}$
Neural Network	Weights W	$V : \mathbb{R}^3$		$\sum_x V(\hat{x})$
Mixture of Gaussians	A_j, c_j, σ_j	$V : \mathbb{R}^3$ With analytical integration, $\int V(.,z)dz : \mathbb{R}^2$	$c_j \to \hat{c}_j = Rc_j + \mathbf{t}$ $V \to \hat{V}$	$\int \hat{V}(y,z)dz$ for $y \in \mathbb{R}^2$

Table 1. Differentiable parameters and operations realized in the generative model, depending of the volume parametrization. V can be equivalently replace with $\tilde{V}$ in Fourier space. Using an interpolation kernel transforms the function V defined on $\{1,\dots,D\}^3$ into $\hat{V}$ defined on $\mathbb{R}^3$.

Appendix C: Inference and ELBO Computations

This appendix provides more details on the Bayesian formulation of the cryoEM recovery problem, and on the corresponding methods. As a reminder, in the main text, we argue that the lower bounds $\mathcal{L}(q,X,\theta)$ are found by showing that, for any probability distribution q_i on the variables h_i, the observed log-likelihood can be written as the sum of two terms:

$$L(X,\theta) = \mathcal{L}(q,X,\theta) + \sum_{i=1}^{n} \mathrm{KL}(q_i(h_i) \parallel p_\theta(h_i|x_i)) = \sum_{i=1}^{n} \left[\mathcal{L}_i(q_i,x_i,\theta) + \mathrm{KL}(q_i(h_i) \parallel p_\theta(h_i|x_i)) \right] \tag{2}$$

where KL is the Kullback-Leibler divergence (KL) defined as $KL(q \parallel p) = \int q(x) \log \frac{q(x)}{p(x)} dx$, and the terms $\mathcal{L}_i$ write:

$$\mathcal{L}_i(q_i,x_i,\theta) = \int_{h_i} q_i(h_i) \log p_\theta(x_i|h_i) dh_i - \mathrm{KL}\left(q_i(h_i) \parallel p_\theta(h_i)\right). \tag{3}$$

The objective of this appendix is to derive these bounds and present the corresponding variational techniques in greater details.

C.1 A Bayesian take on cryo-EM

Before we show these bounds, let us begin by a preliminary remark regarding the model that we are trying to fit. In our formulation in the main text, the parameters θ are considered as unknown, fixed values. Alternatively, one could also try and incorporate the uncertainty of these parameters by adopting a hierarchical approach, and modelling the parameters θ as random variables with prior $p(\theta)$. In this case, inference seeks to estimate θ through the maximum of its posterior distribution $p(\theta|x_1,...,x_n)$, written in its logarithm form as:

$$\log p(\theta|x_1,...,x_n) \propto \log p(\theta,x_1,...,x_n) = \log p_\theta(x_1,...,x_n) + \log p(\theta) = L(X,\theta) + \log p(\theta). \tag{4}$$

This can increase robustness by embedding in the design of the algorithm the inherent variability and uncertainty associated with θ. In fact, following Scheres[13], adding priors on θ can be viewed as a kind of regularization. Such approaches — referred to as Maximum a Posteriori (MAP) approaches — are chosen in Relion-Refine3D and Relion-Class3D[29], FSTdiff[39], and CryoSPARC[16]. In the original RELION[13] method for instance, the underling volume is represented through its Fourier components V_l, whose prior is chosen to be zero-mean Gaussian distributed with unknown variance τ_l^2.

We observe that the MAP objective in Equation (4) only differ from the ML objective through the prior term $\log p(\theta)$, while the challenge of the optimization stems from the intractable term $L(X,\theta)$. Both approaches traditionally employ the same optimization techniques.

C.2 ELBO Computations

We provide the derivations leading to Equations (2)-(3) and the definition of the ELBO as a lower bound to the log-likelihood of the generative model. We start by expressing the observed log-likelihood of the model as:

$$
\begin{aligned}
L(X,\theta) &= \sum_{i=1}^{n} \log p(x_i|\theta) \\
&= \sum_{i=1}^{n} (\log p(x_i,h_i|\theta) - \log p(x_i,h_i|\theta) + \log p(x_i|\theta)) \\
&= \sum_{i=1}^{n} (\log p(x_i,h_i|\theta) - \log p(h_i|x_i,\theta)) \qquad (\text{ Bayes rule:} \quad p(h_i|x_i,\theta) = \frac{p(x_i,h_i|\theta)}{p(x_i|\theta)})
\end{aligned}
$$

In what follows, we drop the index i and associated sum for convenience. Consider any distribution $q(z)$. If we multiply both sides of the above equation by q, and integrate the z out, we get:

$$
\begin{aligned}
&\int_z L(X,\theta) q(h) dh = \int_h (\log p(x,h|\theta) - \log p(h|x,\theta))\, q(h) dh \\
&\implies L(X,\theta) = \int_h (\log p(x,h|\theta) - \log p(h|x,\theta))\, q(h) dh,
\end{aligned}
$$

since $L(X,\theta)$ does not depend on h. Now expanding the right hand side:

$$
\begin{aligned}
L(X,\theta) &= \int_h q(h) \log p(x,h|\theta) dh - \int_h q(h) \log p(h|x,\theta) dh \\
&= \int_h q(h) \log p(x,h|\theta) dh - \int_h q(h) \log \frac{p(h|x,\theta) q(h)}{q(h)} dh \\
&= \int_h q(h) \log p(x,h|\theta) dh - \int_h q(h) \log q(h) dh - \int_h q(h) \log \frac{p(h|x,\theta)}{q(h)} dh \\
&= \int_h q(h) \log p(x,h|\theta) dh - \int_h q(h) \log q(h) dh + KL(q(h) \parallel p(h|x,\theta)) \\
&= \mathbb{E}_q[\log(p_\theta(x,h)] - \int_h q(h) \log q(h) dh + KL(q(h) \parallel p(h|x,\theta)) \\
&= \mathbb{E}_q[\log(p_\theta(x,h)] + H(q) + KL(q(h) \parallel p(h|x,\theta)),
\end{aligned}
$$

by introducing the entropy of q as $H(q) = -\int_z q(z) \log q(z) dz$, and writing the KL-divergence between two distributions p_1 and p_2 as $KL(p_1||p_2) = \int p_1(x) \log(\frac{p_1(x)}{p_2(x)}) dx$.

In other words, for any distribution q on the latent variable H, the observed marginal likelihood $L(X,\theta)$ is the sum of three terms: (a) the expected log-likelihood, assuming that h has distribution q, (b) the Entropy of q, which can be understood of the amount of uncertainty in the estimation of h and (c) the KL-divergence between q and the true posterior $p(h|x,\theta)$, which measures how different these distributions are.

Note that, in the previous equations, terms (a) and (b) can be computed, but term (c) — the KL divergence between the proposed distribution q for h and the actual posterior distribution $p(h|x,\theta)$ is not unknown, because we do not know $p(h|x,\theta)$. However, because the KL-divergence is always non-negative, we know that:

$$L(X,\theta) \geq \mathbb{E}_q[\log(p_\theta(x,h)] + H(q).$$

Thus, introducing the ELBO term $\mathcal{L}(q,X,\theta) = \mathbb{E}_q[\log(p_\theta(x,h)] + H(q)$, we get:

$$L(X,\theta) = \mathcal{L}(q,X,\theta) + KL(q(h) \parallel p(h|x,\theta)). \tag{5}$$

which provides Equation (2) and the right hand side of Equation (3). Developping the ELBO term gives:

$$
\begin{aligned}
\mathcal{L}(q,X,\theta) &= \int_h q(h) \log p(x,h|\theta) dh - \int_h q(h) \log q(h) dh \\
&= \int_h q(h) \log p(x|h,\theta) dh + \int_h q(h) \log p(h|\theta) dh - \int_h q(h) \log q(h) dh \\
&= \int_h q(h) \log p(x|h,\theta) dh - KL(q(h) \parallel p(h|\theta)),
\end{aligned}
$$

which gives the left side of Equation (3).

C.3 Expectation-maximization (EM) Algorithm

Historically, inference in latent variable models has been achieved through the Expectation-maximization (EM) algorithm[41]. The EM algorithm is often introduced as a data imputation technique (see following subsection), but it can also be understood as a dual ascent algorithm — a perspective that allows unifying most cryoEM inference methods and that we present here.

EM as a Dual Ascent Algorithm The EM algorithm can be seen as a dual ascent procedure, *i.e.* as a maximization-maximization procedure, that leverages Equation (2) rewritten as: $\mathcal{L}(q,X,\theta) = L(X,\theta) - \sum_{i=1}^{n} \mathrm{KL}(q_i(h_i) \parallel p_\theta(h_i|x_i))$. Starting with an initial guess $\theta^{(0)}$ of the parameter, the EM algorithm performs two steps at each iteration t:

(a) Inference on hidden variables (E-step) Given the current $\theta^{(t-1)}$, maximize each term $\mathcal{L}_i(q_i,\theta^{(t-1)}) = L_i(X_i,\theta^{(t-1)}) - KL(q_i(h_i)||p_{\theta^{(t-1)}}(h_i,x_i))$ (by Equation 5) with respect to the distribution q_i defined on the hidden variables h_i. Since $\theta^{(t-1)}$ is fixed, the best choice of q is the one that minimizes the KL term $KL(q_i(h_i)||p_{\theta^{(t-1)}}(h_i,x_i))$:

$$\text{For each } i = 1,...,n: \qquad q_i^{(t)}(h_i) = \arg\max_q \mathcal{L}_i(q,X,\theta^{(t-1)}) = \arg\min_q KL(q_i(h_i)||p_{\theta^{(t-1)}}(h_i,x_i)) = p_{\theta^{(t-1)}}(h_i|x_i). \quad (6)$$

This choice $q_i^{(t)}(h_i) = p_{\theta^{(t-1)}}(h_i|x_i)$ corresponds to the green distribution in Figure 2 and makes the lower bound $\mathcal{L}$ tangent to L at $\theta^{(t-1)}$.

(b) Maximization on the model's parameters (M-step) Given the current $q^{(t)} = \{q_1^{(t)},...,q_n^{(t)}\}$, maximize $\mathcal{L}(q^{(t)},X,\theta)$ with respect to the model parameters θ:

$$\theta^{(t)} = \arg\max_\theta \mathcal{L}(q^{(t)},X,\theta) = \arg\max_\theta \sum_{i=1}^{n} \mathcal{L}_i(q_i^{(t)},X,\theta). \quad (7)$$

The argument maximum of $\mathcal{L}$ is used to update $\theta^{(t-1)}$ to $\theta^{(t)}$.

These steps are iterated until convergence in θ. In other words, one first finds the "best" lower bound to $L(X,\theta)$ given $\theta^{(t)}$ by choosing the one that is tangent to $L(X,\theta)$ at $\theta^{(t)}$. Once this bound has been established, we subsequently maximize this bound with respect to the parameter θ. We note that to alleviate potential computational bottleneck in the M step (respectively $\mathcal{L}(q^{(t)},X,\theta)$), the closed form solution can be replaced by simply taking a gradient step. This gives rise to "gradient EM" methods, which we will refer to later, as all methods introduced in the next subsections can be formulated as "gradient" methods too.

Computational Bottleneck: E-step. One of the issues with the EM algorithm relies in the computation of the posterior of the latent variable h_i given x_i and current estimate $\theta^{(t-1)}$:

$$p_{\theta^{(t-1)}}(h_i|x_i) = \frac{p_{\theta^{(t-1)}}(x_i|h_i)p(h_i)}{p_{\theta^{(t-1)}}(x_i)} = \frac{p_{\theta^{(t-1)}}(x_i|h_i)p(h_i)}{\int_{h_i} p_{\theta^{(t-1)}}(x_i,h_i)dh_i}. \quad (8)$$

Unless the posterior can be computed efficiently, this computation can be lengthy. In fact, unless the priors of the different variables are specified using conjugacy — which would constrain the choice of prior distributions that we could consider —, it is difficult to get closed-form updates for the posterior. Alternatively, one can evaluate $p_{\theta^{(t-1)}}(h_i|x_i)$ for each h_i discretized on a grid by computing the integral in Equation (8) via a Riemann sum as in RELION[29] or via Importance Sampling as in CryoSPARC[16]. RELION[29] and CryoSPARC[16]use the EM algorithm with MAP estimation of θ, as opposed to ML estimation.

This discretization remains however a computationally intensive approach. Consequently, despite many advances leveraging GPU computing, the integral in Equation (8) represents the main computational bottleneck in cryo-EM reconstruction methods, and the main reason we might want to look for other, more efficient alternatives.

C.4 Variations on the EM algorithm

We present details on the variations on the EM algorithm discussed in the main text.

C.4.1 Modal EM algorithm

The computational bottleneck observed in the EM algorithm has also motivated the use of approximations in the E-step. The modal approximation of EM amounts to finding a proxy for the posterior $p_{\theta^{(t-1)}}(h_i|x_i)$ in the E-step. This approximation replaces the posterior by its "mode" $\hat{h}_i = \arg\max_h p_{\theta^{(t-1)}}(h|x_i)$, i.e. the most probable $\hat{h}_i$ for each image x_i.

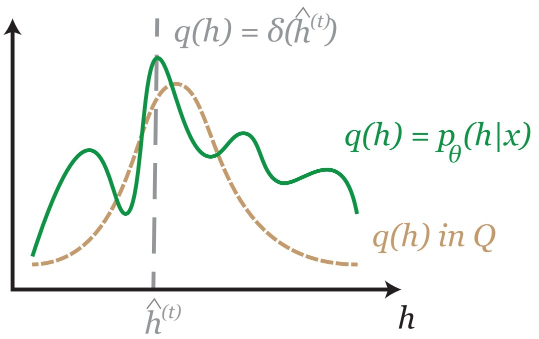

Figure 2. Possible choices for the distribution q on the hidden variables h, where we have dropped the indices i for convenience of notations. The choice of q determines the lower bounds to $L(X,\theta)$. The inference methods used in cryoEM reconstruction can be contrasted by the choice of q: as the true posterior $p_\theta(h|x)$ represented by the green line, as the Dirac distribution $\delta(h)$ represented by the vertical dashed gray line or as a distribution within a known parameterized family $\mathcal{Q}$, such as the family of Gaussian distributions, represented by the brown dashed line.

Modal EM: The algorithm. The E-step of the EM algorithm is replaced by its modal approximation, but the M-step is largely unchanged:

(a) Inference on hidden variables h_i Given current $\theta^{(t)}$, compute the modes:

$$\text{For each } i = 1,...,n: \qquad \hat{h}_i^{(t)} = \arg\max_h p_{\theta^{(t-1)}}(h|x_i) = \arg\max_h p_{\theta^{(t-1)}}(x_i|h)p(h). \tag{9}$$

This amounts to approximating the solution of the E step (which, we've shown, is the posterior of h given X and $\theta^{(t-1)}$) by $q_i^{(t)}(h_i) = \delta(\hat{h}_i^{(t)})$, the Dirac distribution at $\hat{h}_i^{(t)}$, represented in gray in Figure 2. This choice for $q_i^{(t)}(h_i)$ creates a lower bound $\mathcal{L}$ that may not be tangent to L. Since this approximation does not require us to compute complicated posteriors, it is a simpler, but looser ELBO to the observed likelihood.

(b) Maximization on model's parameter θ The parameter θ is updated via:

$$\theta^{(t)} = \arg\max_\theta \sum_{i=1}^n \mathcal{L}(\delta(\hat{h}_i^{(t)}),\theta) = \arg\max_\theta \sum_{i=1}^n p_\theta(x_i,\hat{h}_i^{(t)}). \tag{10}$$

The argument maximum of $\mathcal{L}$ is used to updated $\theta^{(t-1)}$ to $\theta^{(t)}$, just as in the EM algorithm.

Modal EM in RELION[29], CryoSPARC[16], 3DFlex[32], CryoDRGN[23], CryoFold[35] The EM algorithm with modal approximation is used in cryo-EM in CryoSPARC[16], 3DFlex[32], CryoDRGN[23] and CryoFold[35] to infer the latent variable associated with the rotation R_i. In other words, they estimate the best rotation $\hat{R}_i$ for each input image i, relying on a branch-and-bound optimization algorithm to address this maximization. This approach is also used in RELION[29] to estimate the best noise variance $\hat{\sigma}_i$ that is associated with each image i.

C.4.2 Variational EM algorithm

The modal approximation in the EM algorithm speeds up the E-step; yet it has the drawback of summarizing the whole posterior distribution $p_{\theta^{(t)}}(h_i|x_i)$ by a single estimate $\hat{h}_i$, reducing accuracy and leading to non-tangent lower bounds $\mathcal{L}$. Variational Inference (VI), also known as Variational Bayes, has appeared in cryo-EM as a compromise between the guarantees of the EM and the efficiency of modal EM during the computation of the E-step.

Variational Inference (VI) replaces the evaluation of the posterior of the latent variables $p_{\theta^{(t-1)}}(h_i|x_i)$, by an optimizing over a different family of candidate distributions $\mathcal{Q}$, called the "variational family". $\mathcal{Q}$ is typically a family with a restricted form, so that the updates are easier to perform: for instance, if there is a complex dependency structure between entries in our parameter θ, we might select $\mathcal{Q}$ to be a family of posteriors where the entries are independent, so that the updates are simpler to perform: this is usually the 'mean field' approximation to the posterior of θ. The q that is chosen in Equation 3 is the "closest" to the posterior, where "closest" is defined in terms of the KL divergence (see Equation (11) below).

The variational family $\mathcal{Q} = \{q_\eta|\eta\}$ with parameters η is typically chosen to be a family of Gaussian distributions, in which case η represents the mean and variance, i.e. $\mathcal{Q} = \{q_\eta = N(\mu,\sigma)|\eta = (\mu,\sigma)\}$. Other approaches consider the variational family of Gaussian distributions with fixed isotropic variance σ_0, i.e. $\mathcal{Q} = \{q_\eta = \mathcal{N}(\eta,\sigma_0)|\eta\}$. In modal EM, the parametric family can be seen as a family of Dirac distributions, as in Equation (10).

Variational EM as a Dual Ascent Algorithm The E-step of the EM algorithm is replaced by VI and the M-step is unchanged. The VI framework thus gives rise to a variational EM which performs the following two steps at each iteration t:

(a) Inference on hidden variables h_i The n parameters η_i corresponding to each q_i are computed via:

$$\text{For each } i = 1, ..., n: \qquad \eta_i^{(t)} = \arg\min_{\eta} KL(q_\eta || p_{\theta^{(t-1)}}(h_i|x_i)) \tag{11}$$

This choice for q is represented in brown in Figure 2. Similarly to the Modal EM, this creates a lower bound $\mathcal{L}$ that may not be tangent to L.

(b) Maximization on model's parameter θ The parameter θ is updated via:

$$\theta^{(t)} = \arg\max_{\theta} \sum_{i=1}^{n} \mathcal{L}_i(q_{\eta_i}^{(t)}, \theta). \tag{12}$$

The argument maximum of $\mathcal{L}$ is used to updated $\theta^{(t-1)}$ to $\theta^{(t)}$, just as in the EM and the Modal EM algorithms.

Thus, the Variational Inference approximation in the E-step replaces the samplings in evaluation of the true posterior, required to compute the integral in Equation (8), by an optimization on the fewer parameters η_i parametrizing each approximate posterior q_{η_i}. This optimization can be efficiently conducted with (stochastic) gradient descent. In this sense, it also represents a solution to the computational bottleneck of the E-step.

Variational EM in 3DFlex[32] and FSTdiff[39] Variational gradient EM is used in 3DFlex[32] to infer the rotation variable R_i, using the variational family of Gaussian distributions with fixed isotropic variance σ_0.Variational EM is also used in FSTdiff[39]in the context of homogeneous reconstruction. Here, VI happens both in Step (a), to estimate the approximate posterior of the rotation R_i, and in Step (b) to also estimate the approximate posterior of the volume V, which is a parameter included in θ. The approximate posterior of V provides an approximate measure of uncertainty on the homogeneous reconstruction.

Amortized Variational EM as Dual Optimization Algorithm Amortized Inference in the E-step of variational EM leads to an amortized variational EM. In this case, the algorithm can be written as:

(a) Inference on hidden variables h_i The parameters ξ parametrizing the encoder is computed via:

$$\xi^{(t)} = \arg\max_{\xi} \sum_{i=1}^{n} KL(q_{\text{Enc}_\xi(x_i)} || p_{\theta^{(t-1)}}(h_i|x_i)) \tag{13}$$

which generates n distributions $q_{\eta_i}^{(t)}$ parametrized by $\eta_i = \text{Enc}_{\xi^{(t)}}(x_i)$.

(b) Maximization on model's parameter θ The parameter θ is updated via:

$$\theta^{(t)} = \arg\max_{\theta} \mathcal{L}(q_{\eta_i}^{(t)}, \theta). \tag{14}$$

This method has the advantage of further improving the flexibility and efficiency of the Variational EM algorithm, by allowing the updates to be even more efficient by parametrizing the q_i as a function of the input x_i.

C.5 Generative Adversarial Networks

Cryo-EM reconstruction methods from Subsections C.4.1-C.4.2 have naturally explored adaptations of the computationally expensive E-step from the EM algorithm of Subsection C.3. As a result, they differ in their inference on the hidden variables of the generative model. Yet, the only hidden variable of interest is the conformation variable and associated molecular volumes. As a result, recent works have explored methods that avoid the inference on the "nuisance" latent variables altogether. This is the approach taken by Generative Adversarial Network (GAN).

Generative Adversarial Networks as Minimax Games A generative adversarial model (GAN)[43] is a method that estimates the parameters of a generative model, such as the one describing the cryo-EM image formation model, through an adversarial process. The GAN trains a "generator" to produce images x_i that best capture the training data distribution, while a discriminator estimates the probability that a given image x_i came from the training data rather than the generator.

In other words, the generator and discriminator play the following two-player minimax game with value function V:

$$V = \min_{\theta} \max_{\phi} \mathbb{E}_{x \sim p_{\text{data}}(x)} \left[\log \text{Dis}_\phi(x) \right] + \mathbb{E}_{h \sim p(h)} \left[\log \left(1 - \text{Dis}_\phi(f_{V_\theta}(h)) \right) \right],$$

where the generator is the cryo-EM generative model defined in the image formation model and the discriminator has weights ϕ and is denoted by Dis_ϕ. In this equation, $p_{\text{data}}(x)$ represents the probability distribution of the images, and $p(h)$ is a prior distribution on the hidden variables. The GAN training iterates two steps:

(a) Discriminator - Update of parameters ϕ, according to the gradient step:

$$\phi^{(t+1)} = \phi^{(t)} + \nabla_\phi \frac{1}{n} \sum_{i=1}^{n} \left[\log \text{Dis}_\phi(x_i) + \log \left(1 - \text{Dis}_\phi(f_{V_\theta^{(t)}}(h_i)) \right) \right], \tag{15}$$

(b) Generator - Estimation of model's parameter θ, according to the gradient step:

$$\theta^{(t+1)} = \theta^{(t)} - \nabla_\theta \frac{1}{n} \sum_{i=1}^{n} \left[\log \left(1 - \text{Dis}_{\phi^{(t)}}(f_{V_\theta}(h_i)) \right) \right], \tag{16}$$

where in each Step (a) or (b), the "hidden variables" h_i are sampled according to a prior distribution p_h. Even though a GAN iterates two steps, including one step related to the estimation of the model's parameters, its framework differ from the variations of the EM algorithm in the sense that the hidden variables are not inferred: it is enough to be able to randomly sample from them from some prior distribution.

GANs in CryoGAN[38] and Multi-CryoGAN[44] CryoGAN[38] uses a GAN to perform homogeneous reconstruction of molecular volumes, while using uniform sampling on the rotation, translation and CTF hidden variables. Multi-CryoGAN[44] introduces heterogeneous reconstruction with this approach. Both CryoGAN[38] and Multi-CryoGAN[44] use the Wasserstein variant[53] of the traditional GANs.

Appendix D: Details on (Variational) Autoencoders

Autoencoders (AEs) and variational autoencoders (VAEs) are the main realizations of the amortized variational inference approaches in the cryo-EM reconstruction literature. This appendix provides additional details to link the traditional presentations of the AEs and VAEs to the framework described in the main text.

D.1 Autoencoders

An autoencoder traditionally aims to minimize the following loss function:

$$\ell(\theta, \xi) = \sum_{i=1}^{n} ||x_i - \hat{x}_i||^2 = \sum_{i=1}^{n} ||x_i - f_{V_\theta}(h_i)||^2 = \sum_{i=1}^{n} ||x_i - f_{V_\theta}(\text{Enc}_\xi(x_i)||^2, \tag{17}$$

where f_{V_θ} denotes here the output of the generative model (such as the one in the main text) and is usually called a reconstruction: $\hat{x}_i = f_{V_\theta}(h_i)$, while $\ell(\theta, \xi)$ is called the reconstruction loss. Note that the mean square error is used to quantify the reconstruction loss, but it can be replaced by other metrics. For example, the binary-cross entropy is a metric traditionally used to compare images x_i and $\hat{x}_i$. In this context, the goal is to perform the double minization in θ and ξ such that:

$$\hat{\theta}, \hat{\xi} = \arg\min_{\theta, \xi} \ell(\theta, \xi). \tag{18}$$

By considering the decoder as latent variable model: $\hat{x}_i = f_{V_\theta}(h_i) + \varepsilon_i$ with ε_i a standard Gaussian noise, this loss corresponds to the negative log-likelihood, informed by the encoder.

To minimize this objective, the AE takes a gradient step at each iteration t, or backward pass through the network, such that:

(a) Encoder - Inference on latent variables h_i The encoder updates its weight ξ through a gradient step with learning rate α:

$$\xi^{(t)} = \xi^{(t-1)} - \alpha \nabla_\xi \ell(\theta, \xi). \tag{19}$$

(b) Decoder - Estimation of model's parameter θ The decoder updates its weight θ through a gradient step with learning rate α:

$$\theta^{(t)} = \theta^{(t-1)} - \alpha \nabla_\theta \ell(\theta, \xi). \tag{20}$$

We note that this gradient descent is usually performed via stochastic gradient descent, such that only a mini-batch of the data is considered at each iteration to compute ℓ, as opposed to the full dataset of n images.

D.2 Variational Autoencoders

A variational autoencoder traditionally aims to minimize the following loss function, which is the negative ELBO[48]:

$$\mathcal{L}(\xi,\theta) = \sum_{i=1}^{n} \mathbb{E}_{q_\xi(h_i|X_i))}(\log(p_\theta(X_i|z)) + KL(q_\xi(h_i|X_i)||p(h_i)). \tag{21}$$

In this loss, the first term is called the reconstruction term, estimated with one Monte Carlo sample through the so-called "reparametrization trick", and is akin to the reconstruction loss of the AE. The second term is a KL divergence term that is called the regularization term, as it regularizes the posterior of the latent variable h_i by forcing it to be close to the prior $p(h_i)$ of h_i which is modeled by a standard Gaussian distribution.

We can rewrite this loss by using the functions Enc_ξ and Dec_θ, and assuming that the Monte Carlo sampling performed to compute the expectation happens by sampling a unique $\tilde{h}_i$ through $q_\xi(h_i|X_i)$ which is the convention adopted in these architectures:

$$\mathcal{L}(\xi,\theta) = \sum_{i=1}^{n} ||x_i - \hat{x}_i||^2 - KL(q_\xi(h_i|x_i) \parallel p(h_i)) \quad = \sum_{i=1}^{n} ||x_i - \text{Dec}_\theta(\text{Enc}_\xi(x_i)||^2 - KL(q_{\text{Enc}_\xi}(h_i|x_i) \parallel p(h_i)), \tag{22}$$

where the KL term has a closed form in terms of the output of the encoder, due to the fact that the q distribution belongs to a Gaussian family of diagonal covariance.

By realizing that the decoder only participates in the first term of the loss function, the VAE takes a gradient step at each iteration t, or backward pass through the network, such that:

(a) Encoder - Inference on latent variables h_i The encoder updates its weight ξ through a gradient step of learning rate α such that:

$$\xi^{(t)} = \xi^{(t-1)} - \alpha\nabla_\xi\mathcal{L}(\xi,\theta). \tag{23}$$

(b) Decoder - Estimation of model's parameter θ The decoder updates its weight θ through a gradient step with learning rate α:

$$\theta^{(t)} = \theta^{(t-1)} - \alpha\nabla_\theta\mathcal{L}(\xi,\theta) = \theta^{(t-1)} - \alpha\nabla_\theta\ell(\theta,\xi). \tag{24}$$

The main difference with the autoencoder is that the latent variable h_i is considered as a random variable, rather than a fixed deterministic value. That is, h_i is endowed with a parametric probability distribution represented by q — which is a Gaussian distribution with parameters output by the encoder, so that $h_i \sim N(\mu_\xi(X_i), \sigma_\xi^2(X_i))$ and $\text{Enc}_\xi(X_i) = (\mu_x i(X_i), \sigma_\xi^2(X_i)$. Considering h_is as random variables has been shown to lead superior reconstruction results over the autoencoder.

The VAE loss can be adapted as: $\mathcal{L}(\xi,\theta) = \sum_{i=1}^{n} \mathbb{E}_{q_\xi(z|X_i))}(\log(p(X_i|z)) + \beta KL(q_\xi(X_i|Z||p(x))$, where β is an additional hyperparameter introduced in β-VAE[56]. Traditional VAEs have $\beta = 1$, to ensure that the actual negative ELBO is minimized. Yet, to prevent pathological issues in the fitting of VAEs (including posterior collapse), recent work has shown that counterbalancing the reconstruction error with the KL divergence through a β could yield superior results.

In the cryo-EM implementations of the VAE architectures, the ELBO loss can be supplemented with additional terms that we name "structure losses" for now. We will explain it in the next appendix as it depends on the structure of the decoder (i.e. exact choice of generative model) chosen by the method.

In practive, CryoPoseNet[34], CryoAI[18]and E2GMM[33] use an autoencoder, and E2GMM[33] additionally implements an variational autoencoder with a variational family of Gaussian distributions with fixed isotropic variance σ_0. The AE architecture is used in CryoPoseNet[34]with a traditional L2 reconstruction loss, in CryoAI[18]with a "symmetrized" L2 reconstruction loss and in E2GMM[33] with a tailored reconstruction loss that relies on the Fourier ring correlation (FRC) reconstruction metric.

CryoVAEGAN[42] , CryoDRGN[23] , CryoFold[35] , and AtomVAE[17] use VAEs with a variational family of Gaussian distributions with diagonal covariance matrix, to respectively infer (R_i, CTF_i) (CryoVAEGAN), z_i (CryoDRGN and CryoFold) and (z_i, R_i) (atomVAE) – see Table 4.

The VAE architecture is used in CryoDRGN[23] with the negative ELBO loss, and in CryoVAEGAN[42] with the negative ELBO loss extended with the β hyper-parameter described in the supplementary materials, an additional geometric regularization term, while the reconstruction loss relies on the binary cross-entropy as opposed to the L2 reconstruction loss. AtomVAE[17] also implements a VAE with a modified ELBO loss that relies on important sampling (not detailed in this review), that leverages the β hyperparameter and additionally includes a "structure loss" — see supplementary materials.

Appendix E: Constraints

As described in the introduction and following the exposition by Scheres[13] (2012), the molecule reconstruction problem is a difficult, highly non-linear inverse problem, which makes the parametrization of the shape and associated constraints particularly important. This parametrization amounts to impose structure on the desired reconstructed shapes — either by leveraging domain knowledge on the properties of molecular volumes, or by using external information to guide the reconstruction.

From a physics/biology perspective, this can be seen as a necessary enrichment of the cryo-EM data with either external assumptions and/or domain knowledge on the properties of the solution to ensure a more accurate recovery. *From a statistics perspective*, formulated this way, the problem rapidly takes on a Bayesian flavour, and the objective of this step is to find the right “prior" on the distribution of our latent variables. *From a computational perspective*, this corresponds to adding additional terms to the loss/objective defining the optimization problem, and effectively explains what we term “structure loss" in the main text. We detail here the additional constraints that can equip the inference methods across the reconstruction algorithms.

E.1 Smoothness

Smoothness refers to the property by which a signal, or quantity of interest, varies with "no abrupt change" over continuous regions. As explained by Scheres (2012)[13], "because macromolecules consist of atoms that are connected through chemical bonds, the scattering potential will vary smoothly in space, especially at less than atomic resolution." Smoothness of the recovered scattering potential V thus appears to be a reasonable assumption, which is implemented in different ways depending on the parametrization chosen:

Smoothness of the 3D image. The smoothness assumption holds in image space as the image is a projection of the electron field — which is itself continuous. This smoothness is encoded by the normal distribution. This also calls to mind the "ridge penalty", a similar type of regularisation in statistics.

Smoothness of the Fourier coefficients. The smoothness of the 3D density map translates into smoothness over neighbouring Fourier coefficients. In a Bayesian pipeline, this is typically parametrized by assuming that these coefficients are sampled from a normal distribution. The RELION algorithm (Scheres[13]) is based on such a smoothness assumption. This prior is encoded by assuming independent Gaussian priors on the Fourier components of the signal: $V_l \sim N(0, \tau_l^2)$. Note here that the algorithm is not encouraging any other type of structure (e.g sparsity through spike-and-slab prior, etc).

Smoothness of the deformation field. 3DFlex[32] directly exploits the knowledge that conformational variability of a protein is the result of physical processes that transport density over space. This means that mass and local geometry are preserved. As a result, this method implements a convection operator that outputs the deformation field. 3DFlex exploits prior knowledge of smoothness and local rigidity in the deformation field.

Smoothness of the function over 3D coordinates in Fourier domain. Cryo-DRGN[23] and Cryo-Fold[35] represent the volume as the function $f : \Omega^3 \to \mathbb{R}$ over a 3D domain. In practice, this function is implemented by a neural network, which constrains it to be continuous, and possibly smooth if the activation functions used by the network are themselves smooth (sigmoids, for example).

E.2 Rigidity

Other constraints exploit physical properties and knowledge of the system to constrain the reconstruction. Specifically, due to the fact that the molecules studied in cryo-EM are frozen, we can assume that their conformational heterogeneity will not present large variations. As a result, we can assume that the molecular structure has some type of “rigidity". This is implemented in practice by refraining the variables describing the shape heterogeneity from varying excessively. Depending on the parametrization chosen to represent the volume heterogeneity, the rigidity constraint takes different forms:

Rigidity of the deformation field. The deformation field that parametrizes volume heterogeneity in 3D-Flex[32] can be constrained to only generate “small" deformation, through a regularization term. This is integrated as a “structure loss" of the deep learning training procedure of 3D-Flex[32].

Rigidity in (pseudo)-atoms coordinates. The (pseudo-) atoms coordinates that parametrize the volume heterogeneity in Cryo-Fold[35] are constrained to be close to a base conformation’s coordinates, via an $L2$ penalization on the deformations. This forms the “structure loss" integrated in the objective function for the training procedure.

Rigidity in deviation from (pseudo)-atoms coordinates. Here, the volume is parametrized as a set of deviations of pseudo-atoms coordinates, compared to a base conformation, as in Cryo-DeepMind[17]. The rigidity of the molecular structure is thus enforced by adding a constraint that takes the form of a L2 regularization constraining the Δc_j to be small, and effectively forming the “structure loss" added to the optimization objective of this approach.

	Biomolecule	# of images	Image size	Data Type	Noise
CryoDRGN[23]	Ribosome 80S (EMPIAR-10028)	50,000	128 x 128	Synthetic	No/Yes
		105,247	90 x 90	Experimental	Yes
	Ribosome 50S (EMPIAR-10076)	131,899	90 x 90	Experimental	Yes
	Protein complex	50,000	64 x 64	Synthetic	N/A
CryoFold[35]	Haemoglobin (PDB 5NI1)	50,000	128 x 128	Synthetic	Yes
3DFlex[32]	Tri-snRNP spliceosome (EMPIAR-10073)	102,500	180 x 180	Experimental	Yes
	TRPV1 ion-channel (EMPIAR-10059)	200,000	128 x 128	Experimental	Yes
CryoPoseNet[34]	E.coli adenylate kinase (PDB 4AKE1)	9,000	128 x 128	Synthetic	No/Yes
E2GMM[33]	Ribosome 50S (EMPIAR-10076)	124,900	N/A	Experimental	Yes
	Precatalytic spliceosome (EMPIAR-10180)	327,490	N/A	Experimental	Yes
	SARS-CoV-2 spike protein (EMPIAR-10492)	55,159	N/A	Experimental	Yes
FSTdiff[39]	GroEL-GroES protein	40,000	128 x 128	Synthetic	Yes
CryoVAEGAN[42]	Ribosome 80S (EMPIAR-10028)	2, 544	128 x 128	Synthetic	No
		5,119 - 8,278 - 4,917	128 x 128	Experimental	Yes
AtomVAE[17]	Aurora A Kinase (Simulated)	63,000	64 x 64	Synthetic	Yes
CryoAI[18]	80S ribosome (PDB 3J79 and 3J7A)	1,000,000	128 x 128	Synthetic	No/Yes
	SARS-CoV-2 spike protein (PDB 6VYB)	1,000,000	128 x 128	Synthetic	No/Yes
	Spliceosome (PDB 5NRL)	1,000,000	128 x 128	Synthetic	No/Yes
	80S ribosome (EMPIAR-10028)	105,247	256 x 256	Experimental	Yes
CryoGAN[38]	β-galactosidase	41,000	180 x 180	Synthetic	Yes
	β-galactosidase (EMPIAR-10061)	41,123	192 x 192	Experimental	Yes
Multi-CryoGAN[44]	Heat-shock protein Hsp90	100,000	32 x 32	Synthetic	Yes

Table 2. Summary of synthetic and experimental cryo-EM datasets on which the reconstruction methods have been tested. The light red background indicates methods that perform homogeneous reconstruction, and the light yellow background heterogeneous reconstructions.

We also note that the methods that do not assume any heterogeneity in volumes V_i but rather model the volume as a unique possible conformation V, are essentially relying on the rigidity assumption, assuming that variations around V are not large.

Appendix F: Experiments

This appendix summarizes the experiments conducted in the papers cited in this review, in Table 2. As mentioned in the main text, we observe a great diversity of datasets, which explains the difficulty encountered in comparing methods' performances and accuracies.

Appendix G: Classification of Reconstruction Methods

This last appendix provides details in the classification of the reconstruction methods, in the form of two tables. Table 3 compares the generative models and Table 4 compares the inference methods.

References

1. Slavica Jonić Computational methods for analyzing conformational variability of macromolecular complexes from cryo-electron microscopy images. *Curr. Opin. Struct. Biol.* DOI: 10.1016/j.sbi.2016.12.011 (2017).
2. Noe, F. and Clementi C. ollective variables for the study of long-time kinetics from molecular trajectories: theory and methods *Curr. Opin. Struct. Biol.* DOI: 10.1016/j.sbi.2017.02.006 (2017).
3. Katsevich, E. and Katsevich, A. and Singer, A. Covariance Matrix Estimation for the Cryo-EM Heterogeneity Problem *SIAM J. on Imaging Sci.* DOI: 10.1137/130935434 (2015).
4. Haselbach et al Structure and Conformational Dynamics of the Human Spliceosomal Bact Complex *Cell* DOI: 10.1016/j.cell.2018.01.010 (2018).
5. Bock, Lars V. and Grubmüller, Helmut Effects of cryo-EM cooling on structural ensembles *BioRxiv* DOI: 10.1101/2021.10.08.463658 (2021).
6. Noé, Frank and Tkatchenko, Alexandre and Müller, Klaus-Robert and Clementi, Cecilia Machine Learning for Molecular Simulation *Annu. Rev. Phys. Chem.* DOI: 10.1146/annurev-physchem-042018-052331 (2020).

Volume Param.		Space	Conformational Model	Approach	Hidden Variable	Known Variable	Reference Volume
**Discrete Representation**	Voxel Grid	Image	Homogeneous<br>$z \longrightarrow V$	CryoPoseNet[34]	-<br>Rotation<br>-	CTF<br>-<br>Translation	Free
				CryoGAN[38]	-<br>Rotation<br>Translation	CTF<br>-<br>-	Free
			Heterogeneous<br>$z \longrightarrow V$	Multi-CryoGAN[44]	-<br>Rotation<br>Translation	CTF<br>-<br>-	Free
			Heterogeneous<br>$z \longrightarrow f(U(z_j), V_0)$	3DFlex[32]	-<br>-<br>-	CTF<br>Rotation<br>Translation	Yes
		Fourier	Homogeneous<br>$z = (\mu_V, \sigma_V) \longrightarrow V$	FSTdiff[39]	-<br>Rotation<br>Translation	CTF<br>-<br>-	Free
			Heterogeneous<br>$z \in \{1, .., K\} \longrightarrow \{V_1, ..., V_K\}$	RELION[29]	-<br>Rotation<br>Translation	CTF<br>-<br>-	Free
			Heterogeneous<br>$z \in \{1, .., K\} \longrightarrow \{V_1, ..., V_K\}$	CryoSPARC[16]	-<br>Rotation<br>Translation	CTF<br>-<br>-	Free
**Continuous Field**	Neural Network	Image	Homogeneous<br>$z = (\mu_V, \sigma_V, CTF_V) \longrightarrow V_i(2D)$	CryoVAEGAN[42]	CTF<br>2D Rotation<br>-	-<br>-<br>Translation	Free
		Fourier	Heterogeneous<br>$z \longrightarrow V_j$	CryoDRGN[23]	-<br>Rotation<br>Translation	CTF<br>-<br>-	Free
			Homogeneous<br>$z \longrightarrow V_j$	CryoAI[18]	-<br>Rotation<br>Translation	CTF<br>-<br>-	Free
	Gaussian Mixture	Image	Heterogeneous<br>$z \longrightarrow \{c_j, A_j, \sigma_j\}_{j \in [1,N]}$ or $\{\Delta c_j\}_{j \in [1,N]}$; (N "blobs")	E2GMM[33]	-<br>-<br>-	CTF<br>Rotation<br>Translation	Free or Yes
			Heterogeneous<br>$z \longrightarrow \{c_j\}_{j \in [1,N]}$; ($N$ residues)	CryoFold[35]	-<br>-<br>-	CTF<br>Rotation<br>Translation	Free
			Heterogeneous<br>$z \longrightarrow \{\Delta c_j\}_{j \in [1,N]}$; ($N$ residues)	AtomVAE[17]	-<br>Rotation<br>Translation	CTF<br>-<br>-	Yes

Table 3. Classification of reconstruction methods in terms of the parametrization of the volume V (or $\tilde{V}$), *i.e.* the model of conformational heterogeneity with the conformation variable z, use of image versus Fourier space, representation as a discrete or continuous field, and with a reference-free or reference-based encoding. We also indicate whether nuisance variables are hidden or assumed to be known in the reconstruction methods cited. When not specified, the latent variable z belongs to a vector space $\mathbb{R}^L$.

7. Ourmazd, A. Cryo-em, XFELs and the structure conundrum in structural biology. *Nat. Methods* DOI: 10.1038/s41592-019-0587-4 (2019).

8. Nakane, T. *et al.* Single-particle cryo-em at atomic resolution. *Nature* **587**, 152–156 (2020).

9. EMDB. Statistics. https://www.ebi.ac.uk/emdb/statistics/emdb_resolution_year (2022).

10. Dill, Ken A & et al. Molecular driving forces: statistical thermodynamics in biology, chemistry, physics, and nanoscience. In *Garland Science* (2010).

11. Kohl, H. & Reimer, L. *Transmission Electron Microscopy*. Springer Series in Optical Sciences.

12. Vulović, M. *et al.* Image formation modeling in cryo-electron microscopy. *J. Struct. Biol.* **183**, 19–32, DOI: https://doi.org/10.1016/j.jsb.2013.05.008 (2013).

13. Scheres, S. H. RELION: Implementation of a Bayesian approach to cryo-EM structure determination. *J. Struct. Biol.* DOI: 10.1016/j.jsb.2012.09.006 (2012).

14. Singer, A. & Sigworth, F. J. Computational methods for single-particle electron cryomicroscopy. *Annu. Rev. Biomed. Data Sci.* **3**, 163–190 (2020).

	**Posterior** $p(h_i \mid x_i)$	**Mode** $\hat{h}_i$		**Variational** $q(h_i)$	
		**Non-Amortized**	**Amortized (encoder)**	**Non-Amortized**	**Amortized (encoder)**
Distribution on h_i	$p(h_i \mid x_i)$	$\arg\max_z p(h_i \mid x_i)$	$\mathrm{Enc}_\xi(x_i)$	$q_{\eta_i} \in \mathcal{Q}$	$q_{\mathrm{Enc}_\xi(x_i)} \in \mathcal{Q}$
**Name**	**EM**	**Approx. EM**	**Am. EM / AE**	**Var. EM / AD**	**Var. Am. EM / VAE**
Inference on h_i (E-step)	RELION[13] (pose R_i, t_i and conformation z_i)	RELION[13] (noise σ_i) (*)	CryoPoseNet[34] (rotation R_i)	3DFlex[32] (conformation z_i)	CryoDRGN[23] (conformation z_i)
		CryoSPARC[16] (rotation R_i) (*)	E2GMM[33] (conformation z_i)	FSTdiff[39] (pose R_i, t_i and conformation z)	CryoVAEGAN[42] (2D rotation R_i and CTF_i)
		CryoDRGN[23] (pose R_i, t_i) (*)	CryoAI[18] (pose R_i, t_i)		atomVAE[17] (pose R_i, t_i and conformation z_i)
		3DFlex[32] (conformation z_i)			CryoFold[35] (conformation z_i)

Table 4. Classification of cryo-EM reconstruction algorithms with respect to the inference on the conformation z_i and on the unknown hidden variables h_i. The inference step updates the posterior $p(h_i|x_i$, or its mode $\hat{h}_i$, or a variational approximation $q(h_i)$ of it — which corresponds to the 3 main columns of the table. The works by Zhong et al. (2019, 2021) are classified in several cells, as inference of the conformation z_i is performed via a VAE, but the inference of the poses R_i, t_i is performed with an optimization algorithm. The notation (*) specifies that the optimization associated with this step is fully performed (until convergence to local extremum) while its absence indicates that only a gradient step is taken toward the optimum.

15. Kimanius, D., Forsberg, B. O., Scheres, S. H. & Lindahl, E. Accelerated cryo-EM structure determination with parallelisation using GPUS in RELION-2. *eLife* DOI: 10.7554/eLife.18722 (2016).

16. Punjani, A., Rubinstein, J. L., Fleet, D. J. & Brubaker, M. A. CryoSPARC: Algorithms for rapid unsupervised cryo-EM structure determination. *Nat. Methods* DOI: 10.1038/nmeth.4169 (2017).

17. Rosenbaum, D. *et al.* Inferring a continuous distribution of atom coordinates from cryo-em images using vaes. *CoRR* **abs/2106.14108** (2021). 2106.14108.

18. Veym, A. *et al.* CryoAI: Amortized Inference of Poses for Ab Initio Reconstruction of 3D Molecular Volumes from Real Cryo-EM Images. *arXiv* (2022). 2203.08138.

19. Boyd, S., Boyd, S. P. & Vandenberghe, L. *Convex optimization* (Cambridge university press, 2004).

20. Bendory, T., Bartesaghi, A. & Singer, A. Single-particle cryo-electron microscopy: Mathematical theory, computational challenges, and opportunities. *IEEE signal processing magazine* **37**, 58–76 (2020).

21. Sitzmann, V. *et al.* Implicit Neural Representations with Periodic Activation Functions. *arXiv preprint arxiv.2006.09661* (2020).

22. Si, D. *et al.* Artificial intelligence advances for de novo molecular structure modeling in cryo-em. *arXiv preprint arXiv:2102.06125* (2021).

23. Zhong, E. D., Bepler, T., Davis, J. H. & Berger, B. Reconstructing continuously heterogeneous structures from single particle cryo-em with deep generative models. *arXiv preprint arXiv:1909.05215* (2019).

24. Zhong, E. D., Bepler, T., Berger, B.& Davis, J. H. CryoDRGN: reconstruction of heterogeneous cryo-EM structures using neural networks. *Nat. methods* (2021).

25. Zhong, E. D., Lerer, Adam, Davis, J. H. & Berger, B. CryoDRGN2: Ab initio neural reconstruction of 3D protein structures from real cryo-EM images. *Proc. IEEE/CVF Int. Conf. on Comput. Vis.* (2021).

26. Ede, J. M. Deep learning in electron microscopy. *Mach. Learn. Sci. Technol.* **2**, 011004 (2021).

27. Wu, J.-G. *et al.* Machine learning for structure determination in single-particle cryo-electron microscopy: A systematic review. *IEEE Transactions on Neural Networks Learn. Syst.* 1–21, DOI: 10.1109/TNNLS.2021.3131325 (2021).

28. Sharon, N., Kileel, J., Khoo, Y., Landa, B. & Singer, A. Method of moments for 3d single particle ab initio modeling with non-uniform distribution of viewing angles. *Inverse Probl.* **36**, 044003 (2020).

29. Scheres, S. H. A bayesian view on cryo-EM structure determination. *J. Mol. Biol.* DOI: 10.1016/j.jmb.2011.11.010 (2012).

30. Scheres, S. H. Chapter eleven - classification of structural heterogeneity by maximum-likelihood methods. In Jensen, G. J. (ed.) *Cryo-EM, Part B: 3-D Reconstruction*, vol. 482 of *Methods in Enzymology*, 295–320, DOI: https://doi.org/10.1016/S0076-6879(10)82012-9 (Academic Press, 2010).

31. Punjani, A. & Fleet, D. J. 3d variability analysis: Resolving continuous flexibility and discrete heterogeneity from single particle cryo-em. *J. Struct. Biol.* **213**, 107702, DOI: https://doi.org/10.1016/j.jsb.2021.107702 (2021).

32. Punjani, A. & Fleet, D. J. 3d flexible refinement: Structure and motion of flexible proteins from cryo-em. *bioRxiv* DOI: 10.1101/2021.04.22.440893 (2021). https://www.biorxiv.org/content/early/2021/04/22/2021.04.22.440893.full.pdf.

33. Chen, M. & Ludtke, S. J. Deep learning-based mixed-dimensional gaussian mixture model for characterizing variability in cryo-em. *Nat. Methods* **18**, 930–936, DOI: 10.1038/s41592-021-01220-5 (2021).

34. Nashed, Y. S. G. *et al.* End-to-end simultaneous learning of single-particle orientation and 3d map reconstruction from cryo-electron microscopy data (2021). 2107.02958.

35. Zhong, E. D., Lerer, A., Davis, J. H. & Berger, B. Exploring generative atomic models in cryo-em reconstruction (2021). 2107.01331.

36. Rose, Y. *et al.* Rcsb protein data bank: Architectural advances towards integrated searching and efficient access to macromolecular structure data from the pdb archive. *J. Mol. Biol.* **433**, 166704, DOI: https://doi.org/10.1016/j.jmb.2020.11.003 (2021). Computation Resources for Molecular Biology.

37. AlQuraishi, M. Alphafold at casp13. *Bioinformatics* **35**, 4862–4865 (2019).

38. Gupta, H., McCann, M. T., Donati, L. & Unser, M. Cryogan: a new reconstruction paradigm for single-particle cryo-em via deep adversarial learning. *IEEE Transactions on Comput. Imaging* **7**, 759–774 (2021).

39. Ullrich, K., van den Berg, R., Brubaker, M. A., Fleet, D. J. & Welling, M. Differentiable probabilistic models of scientific imaging with the fourier slice theorem. *CoRR* **abs/1906.07582** (2019). 1906.07582.

40. Rohou, A. & Grigorieff, N. CTFFIND4: Fast and accurate defocus estimation from electron micrographs. *J. Struct. Biol.* DOI: 10.1016/j.jsb.2015.08.008 (2015).

41. Dempster, A., Laird, N. & Rubin, D. B. Maximum Likelihood from Incomplete Data via the EM Algorithm. *J. Royal Stat. Soc. Ser. B (Methodological)* **39**, 1–38 (1977).

42. Miolane, N., Poitevin, F., Li, Y.-T. & Holmes, S. Estimation of orientation and camera parameters from cryo-electron microscopy images with variational autoencoders and generative adversarial networks. *CVPR conference, Work. on Comput. Vis. for Microsc. Image Analysis* (2019). arXiv:1911.08121.

43. Goodfellow, I. J. *et al.* Generative Adversarial Nets. Tech. Rep. (2014).

44. Gupta, H., Phan, T. H., Yoo, J. & Unser, M. Multi-cryogan: Reconstruction of continuous conformations in cryo-em using generative adversarial networks. In *European Conference on Computer Vision*, 429–444 (Springer, 2020).

45. Gelfand, A. E. & Smith, A. F. M. Sampling-based approaches to calculating marginal densities. *J. Am. Stat. Assoc.* **85**, 398–409, DOI: 10.1080/01621459.1990.10476213 (1990).

46. Duane, S., Kennedy, A., Pendleton, B. J. & Roweth, D. Hybrid monte carlo. *Phys. Lett. B* **195**, 216–222, DOI: 10.1016/0370-2693(87)91197-x (1987).

47. Henderson, R. Avoiding the pitfalls of single particle cryo-electron microscopy: Einstein from noise. *Proc. Natl. Acad. Sci.* **110**, 18037–18041 (2013).

48. Kingma, D. P. & Welling, M. Auto-Encoding Variational Bayes. In *Proceedings of the 2nd International Conference on Learning Representations (ICLR)* (2014).

49. Marin van Heel & Michael Schatz Fourier shell correlation threshold criteria *J. Struct. Biol.* **151**, 250–262, DOI: 10.1016/j.jsb.2005.05.009 (2005).

50. Harauz G & Marin van Heel Exact filters for general geometry three dimensional reconstruction *Optik* **73**, 146-156, (1986).

51. Lawson, C.L. & et al. Cryo-EM model validation recommendations based on outcomes of the 2019 EMDataResource challenge. *Nat Methods* **18**, 156–164, DOI: 10.1038/s41592-020-01051-w(2021).

52. Renaud, Jean-Paul & et al. Cryo-EM in drug discovery: achievements, limitations and prospects *Nat. reviews Drug discovery* **17**, 471–492, DOI: 10.1038/s41592-020-01051-w(2018).

53. Arjovsky, M. & Welling, M. Wasserstein Generative Adversarial Networks. In *Proceedings of Machine Learning Research* (2017)

54. Deng, Lie The mnist database of handwritten digit images for machine learning research. In *IEEE Signal Processing Magazine* (2012)

55. Deng, Jia & et al. Imagenet: A large-scale hierarchical image database. In *2009 IEEE conference on computer vision and pattern recognition* (2009)

56. Thiagarajan, Ganesh & Voyiadjis, George Z. β-VAE: Learning basic visual concepts with a constrained variational framework. In *Proceedings of ICLR* (2017)

57. Kufareva, Irina & Abagyan, Ruben Methods of protein structure comparison. In *Homology modeling* (2011)

58. Scherer, Martin K & Husic, Brooke E & Hoffmann, Moritz & Paul, Fabian & Wu, Hao & Noé, Frank Variational selection of features for molecular kinetics. In *The Journal of chemical physics* (2019)
